# Genome Wide Methylome Alterations in Lung Cancer

**DOI:** 10.1371/journal.pone.0143826

**Published:** 2015-12-18

**Authors:** Nandita Mullapudi, Bin Ye, Masako Suzuki, Melissa Fazzari, Weiguo Han, Miao K. Shi, Gaby Marquardt, Juan Lin, Tao Wang, Steven Keller, Changcheng Zhu, Joseph D. Locker, Simon D. Spivack

**Affiliations:** 1 Department of Medicine/Pulmonary, Albert Einstein College of Medicine, Bronx, New York, United States of America; 2 Department of Bioinformatics, Albert Einstein College of Medicine, Bronx, New York, United States of America; 3 Department of Genetics, Albert Einstein College of Medicine, Bronx, New York, United States of America; 4 Department of Epidemiology & Population Health, Division of Biostatistics, Albert Einstein College of Medicine, Bronx, New York, United States of America; 5 Department of Epidemiology & Population Health, Albert Einstein College of Medicine, Bronx, New York, United States of America; 6 Department of Cardiovascular &Thoracic Surgery, Montefiore Medical Center, Bronx, New York, United States of America; 7 Department of Pathology, Montefiore Medical Center, Bronx, New York, United States of America; University of Bonn, Institut of Experimental Hematology and Transfusion Medicine, GERMANY

## Abstract

Aberrant cytosine 5-methylation underlies many deregulated elements of cancer. Among paired non-small cell lung cancers (NSCLC), we sought to profile DNA 5-methyl-cytosine features which may underlie genome-wide deregulation. In one of the more dense interrogations of the methylome, we sampled 1.2 million CpG sites from twenty-four NSCLC tumor (T)–non-tumor (NT) pairs using a methylation-sensitive restriction enzyme- based HELP-microarray assay. We found 225,350 differentially methylated (DM) sites in adenocarcinomas *versus* adjacent non-tumor tissue that vary in frequency across genomic compartment, particularly notable in gene bodies (GB; p<2.2E-16). Further, when DM was coupled to differential transcriptome (DE) in the same samples, 37,056 differential loci in adenocarcinoma emerged. Approximately 90% of the DM-DE relationships were non-canonical; for example, promoter DM associated with DE in the same direction. Of the canonical changes noted, promoter (PR) DM loci with reciprocal changes in expression in adenocarcinomas included *HBEGF*, *AGER*, *PTPRM*, *DPT*, *CST1*, *MELK;* DM GB loci with concordant changes in expression included *FOXM1*, *FERMT1*, *SLC7A5*, and *FAP* genes. IPA analyses showed adenocarcinoma-specific promoter DMxDE overlay identified familiar lung cancer nodes [*tP53*, *Akt*] as well as less familiar nodes [*HBEGF*, *NQO1*, *GRK5*, *VWF*, *HPGD*, *CDH5*, *CTNNAL1*, *PTPN13*, *DACH1*, *SMAD6*, *LAMA3*, *AR*]. The unique findings from this study include the discovery of numerous candidate The unique findings from this study include the discovery of numerous candidate methylation sites in both PR and GB regions not previously identified in NSCLC, and many non-canonical relationships to gene expression. These DNA methylation features could potentially be developed as risk or diagnostic biomarkers, or as candidate targets for newer methylation locus-targeted preventive or therapeutic agents.

## Introduction

Lung cancer is responsible for the highest number of cancer-related deaths in the United States [1]. Cancer is characterized by genome-wide changes in CpG methylation, including a generalized genome-wide hypomethylation (loss of methylation) including at oncogenes, and reciprocal hypermethylation at particular loci (increased methylation), including tumor suppressor gene promoters [2,3]. Recent studies have shown that the functional consequence of 5-methylation of cytosine is dependent on the genomic context and specific sequence in which it occurs [4,5]. Methylation of CG residues within CG islands (CGI) in gene promoters is associated with gene silencing. However, methylation of CGI within gene bodies is found to be associated with tissue-specific expression and gene activation in cancer genomes [6–8].

Panels of well-known candidate tumor suppressor genes have been examined in clinical lung cancer specimens to characterize promoter-methylation [9,10], yielding concise methylation signatures [11] as well as to distinguish the different histological sub-types [12]. Methylation changes occur early during the development of lung cancer [13] and thus can be used as predictive markers to detect potential malignancies [14,15]. Thus, the identification of discriminatory methylation marks can be further developed into diagnostic assays to aid in risk assessment and diagnostics.

DNA methylation can be measured by targeted methods such as bisulfite sequencing (tBGS) [16], methylation-specific PCR (MSP) [17], and mass spectrometry-based methods (Epityper^®^) [18]. Each platform assays locus-specific methylation at higher resolution, wherein a defined panel of genes can be assessed for the methylation status of a select number of CpG residues within them. However these methods depend on prior knowledge of specific epigenomic loci to design the assay.

Among discovery methods to detect methylation patterns at a genome-wide scale, one approach is to employ methylation-sensitive and resistant isoschizmer restriction enzymes (HELP, RLM, others). Other approaches include chromatin immunoprecipitation with methylated DNA-binding antibodies (MBD, MeDIP, others), or bisulfite sequencing of a reduced component of the genome (RRBGS, others) [19]. Each of these methods has its own biases and by necessity of scale, samples only a small subset of the human methylome. Whole genome bisulfite sequencing [20] is designed to densely query the entire methylome at single base-specific resolution. However currently this method is too costly and analytically intensive to perform on large sample sizes.

Recent studies have assessed genome-wide methylation in lung cancer to discover tumor specific methylation signatures of cancer genomes [13,21,22]. Selamat *et al* [23] used the Illumina Infinium HumanMethylation27k platform to characterize genome-wide methylation of ~27,000 CpG sites in 59 matched T/NT lung adenocarcinoma samples, and coupled that to transcriptome arrays. Comprehensive molecular profiling of 230 patients (Adenocarcinoma) and 178 patients (Squamous Cell Carcinomas) by TCGA [24,25] made use of an expanded version of the same platform, HM450k, which interrogates more than 480,000 CpG sites, across CpG islands and shores in the human genome.

We hypothesized that an unbiased genome-wide tumor vs non-tumor search for differentially methylated loci will lead to the identification of novel and known loci deregulated in lung cancer. Additionally, investigating the same specimens for differential gene expression would allow identification of higher impact DM loci, by virtue of potential impact on expression. To test these hypotheses, we used the HELP assay [26] to assay the CpG methylation of 24 pairs of tumor (T) and adjacent non-tumor (NT) human samples. This assay queries 1.2 million CCGG motif-defined fragments across the genome by restriction enzymes *Hpa*II (methylation sensitive) and *Msp*I (methylation resistant) to isolate differentially methylated fragments of the genome. These fragments are then adapter-ligated and amplified and labeled, following which they are co-hybridized to a high density microarray. Methylation is detected at ends of enzyme-generated fragments (CCGG sites) and measured as a ratio of *Msp*I-generated fragments to *Hpa*II-generated fragments. Reasoning that methylation-deregulated genes might be more apparent if cognate/proximate gene expression is altered, we further examined the association of differentially methylated (DM) regions with differentially expressed (DE) genes, using mRNA expression data from the same paired T and NT surgical resection samples.

## Results

### Genome-wide survey of differentially methylated loci in lung tumor versus non-tumor

**Fig 1:**
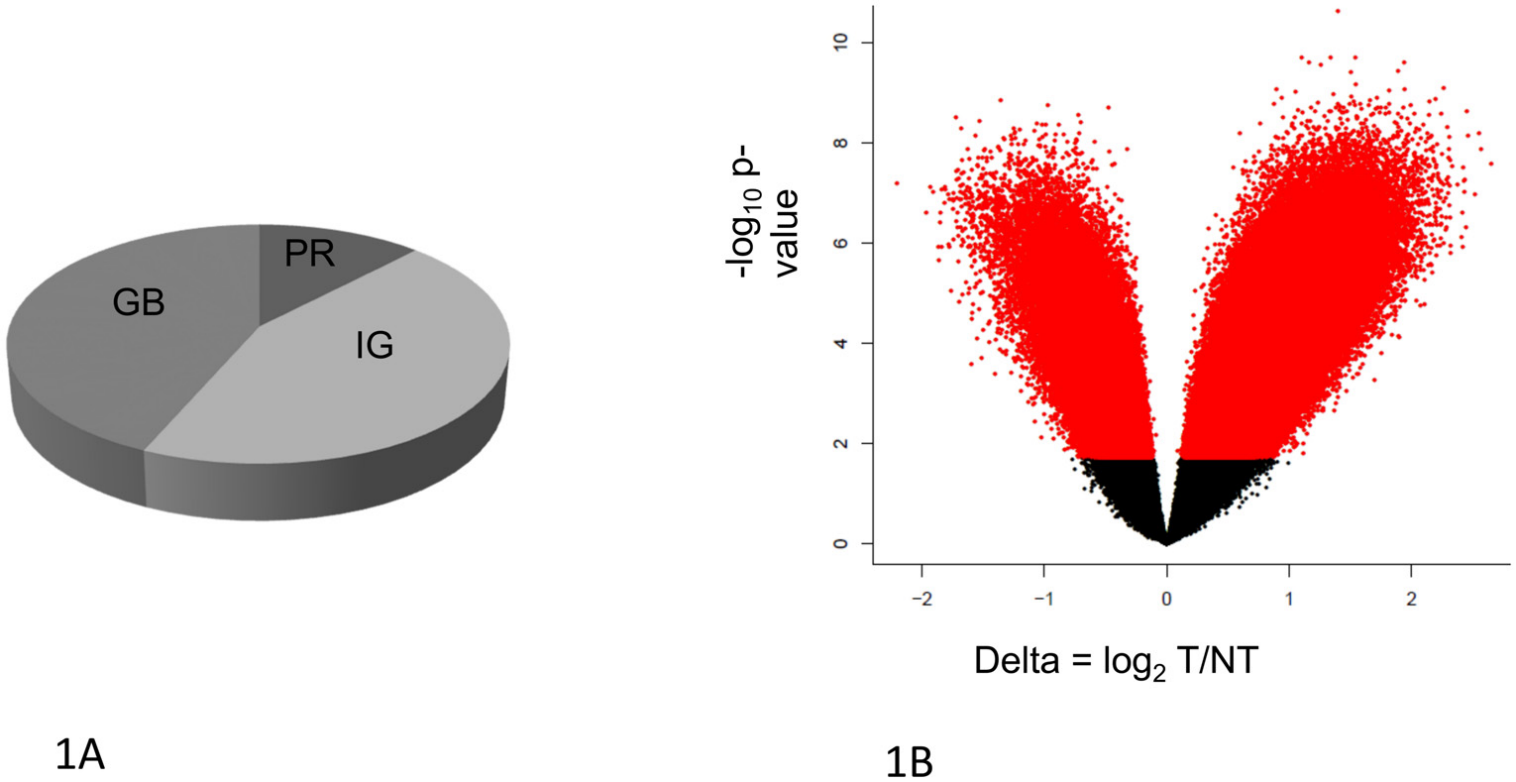
The genome compartment represented on the HELP Nimblegen microarray and statistically significant DM loci. (A) Approximately 91% of the 1.2 million loci represented on the HELP microarray are located in gene body (GB) and intergenic (IG) regions, with a small minority (9%) of the loci located within promoters (PR). (B) Statistical significance (Y-axis) vs. delta (X-axis) (magnitude) of DM. Delta (X-axis) indicates the difference in methylation between tumor (T) vs non-tumor (NT) at a given locus. Loci hypermethylated in T relative to NT have delta < 0. P-value (Y-axis) is calculated based on Benjamini Hochberg adjusted FDR. At FDR p < 0.05, 433,505 loci across all genomic compartments are found to be differentially methylated in T vs NT. Red dots indicate statistically significant DM loci.

Among 24 NSCLC lung resection donors (S1 Table), using the HELP-microarray assay we identified 452,754 HpaII fragments significantly differentially methylated (DM; p<0.05, FDR-adjusted) in tumor versus adjacent non-tumor (Table 1). Of these DM sites, 57% were found in coding regions (comprising 38% of those CCGG sites represented on the array). (Fig 1) Another 39% of these were found in intergenic regions (48% of those sites represented on the array) and were mostly hypomethylated in tumors. Approximately 7% were found in promoter regions (26% of those sites on the array). Gene promoters (PR) and gene bodies (GB) showed both hyper- and hypo-methylation. (Table 1). Promoter hypomethylation exceeded hypermethylation in number (Table 1). Based on a permutation test conducted using random sampling within compartments (PR/GB/IG) we found that DM loci are significantly over-represented in gene body regions (p< 2.2e-16).

**Table 1:** Genomic distribution of DM sites. 452754 loci are significantly differentially methylated (DM) between T and NT based on an FDR < 0.05. Majority of the DM loci are hypomethylated in T vs NT.

Genomic compartment	# loci on array	Differentially methylated loci FDR p< 0.05 (% of loci represented)	Hypomethylated in tumors	Hypermethylated in tumors
Promoters	151568	32037 (26%)	68%	31%
Gene Body regions	551628	248721 (38%)	74%	25%
Intergenic regions	540473	171996 (48%)	93%	6%

**Fig 2:**
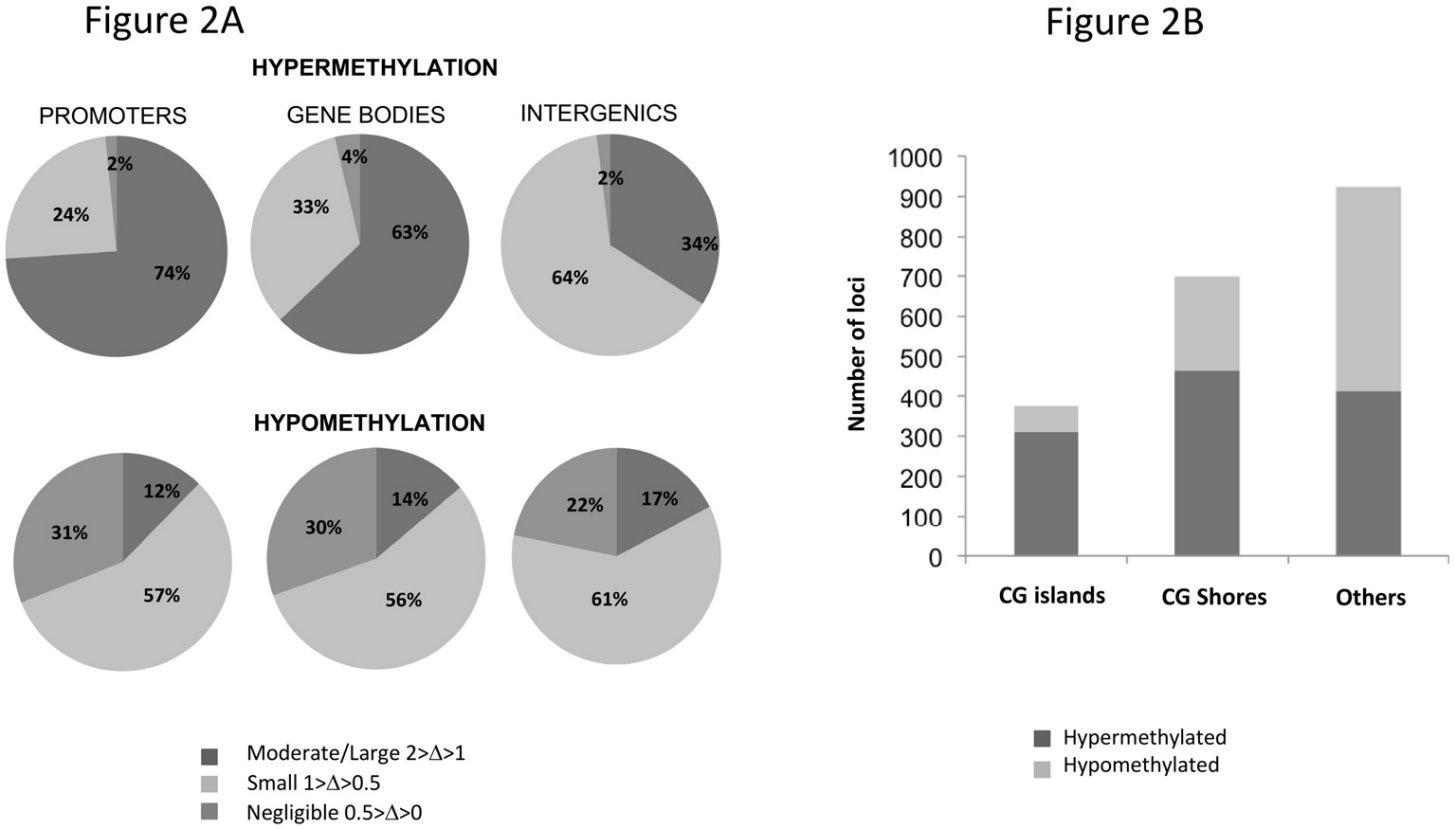
Magnitude and Direction of differential methylation and its distribution across genomic compartments. (A) All NSCLC histologies DM was classified as negligible, small or moderate based on the absolute value. (1< abs delta <2 is Moderate/Large; 1< abs delta <0.5 is Small; 0.5< abs delta <0 is Negligible). DM loci with FDR p<0.05 based on paired T-test were considered for this analysis. Majority of hypermethylation in tumors is observed to be of moderate/large magnitude in promoters and gene bodies, while in the intergenic regions, small changes are most frequent. The majority of hypomethylation is observed to be of small magnitude in all the three compartments. A significant fraction of hypomethylation changes are of negligible magnitude yet statistically significant. (B) Direction of DM and the distribution within promoters categorized based on location within CG-islands and CG-shores. Within the category of DM promoter loci, hypermethylation is more frequent in tumors as compared to hypomethylation for those loci within CG-islands and CG-shores. Overall DM differences do vary by PR genomic location (CGI, CGS, other); all NSCLC histologies were ChiSquare p = 2.2E-16; adenocarcinoma-only histology ChiSquare p = 1.9E-4.

The magnitude of differential methylation (delta) varied by compartment and direction of change. Moderate/large degrees of DM hypermethylation in PR and GB (delta>1; PR = 74%, GB = 63%) were more common than small degrees (1<delta<0.5; PR = 24%, GB = 33%) of hypermethylation changes in these compartments (Fig 2). The magnitude of moderate/large hypermethylation changes were distinct from that of hypomethylation changes, where the moderate/large distribution by genomic region was PR = 12%, GB = 14%, IG = 17%. Within tumor promoters, CG islands (CGI) and CG shores (CGS) were more often hypermethylated than hypomethylated (Fig 2B). Overall distribution of DM loci varied by PR genomic location (CGI, CGS, other) among all NSCLC histologies (ChiSquare p = 2.2E-16) and among adenocarcinoma-only (ChiSquare1.9E-4). There was substantial DM outside of CGI and CGS.

### Individual cancer genes identified by differential methylation

The top 25 differentially methylated loci within promoter regions and gene bodies are listed (S2 Table). In brief, for all histologies combined DM was observed in many promoters (S2A Table) [hypermethylation in *C7orf54*, *DARS*, *SPTAN1*, *DOM3Z*, *PCNX*, *CTNNAL1*, others; hypomethylation in *NQO1*, *SIRP1B*, *UNC5CL*, *NFIA*, *CST1*, others] and in gene bodies [hypermethylation in *NOL10*, *ARHGEF12*, *UST*, *RGS3*, *MBNL2*, others; hypomethylation *FBXL7*, *RYR2*, *NTRK3*, *ADAMTS12*, *PARK2*, others]. For adenocarcinoma specifically, DM was observed in promoters [hypermethylated *RASL12; SPTAN1*, *mir-26a*, hypomethylated *NQO1*, *SIRPB1*, *NF1A*] and gene bodies [hypermethylated *AKAP13*, *ANK family*, *PRKCE*, *ROS1*; hypomethylated *FAM171A1*, *PARK2*, *BCAS3*, *RHOJ*] and many others.

### Heat maps of top 50 most differentially methylated loci within the subset of adenocarcinomas (Fig 3A and 3B)

**Fig 3:**
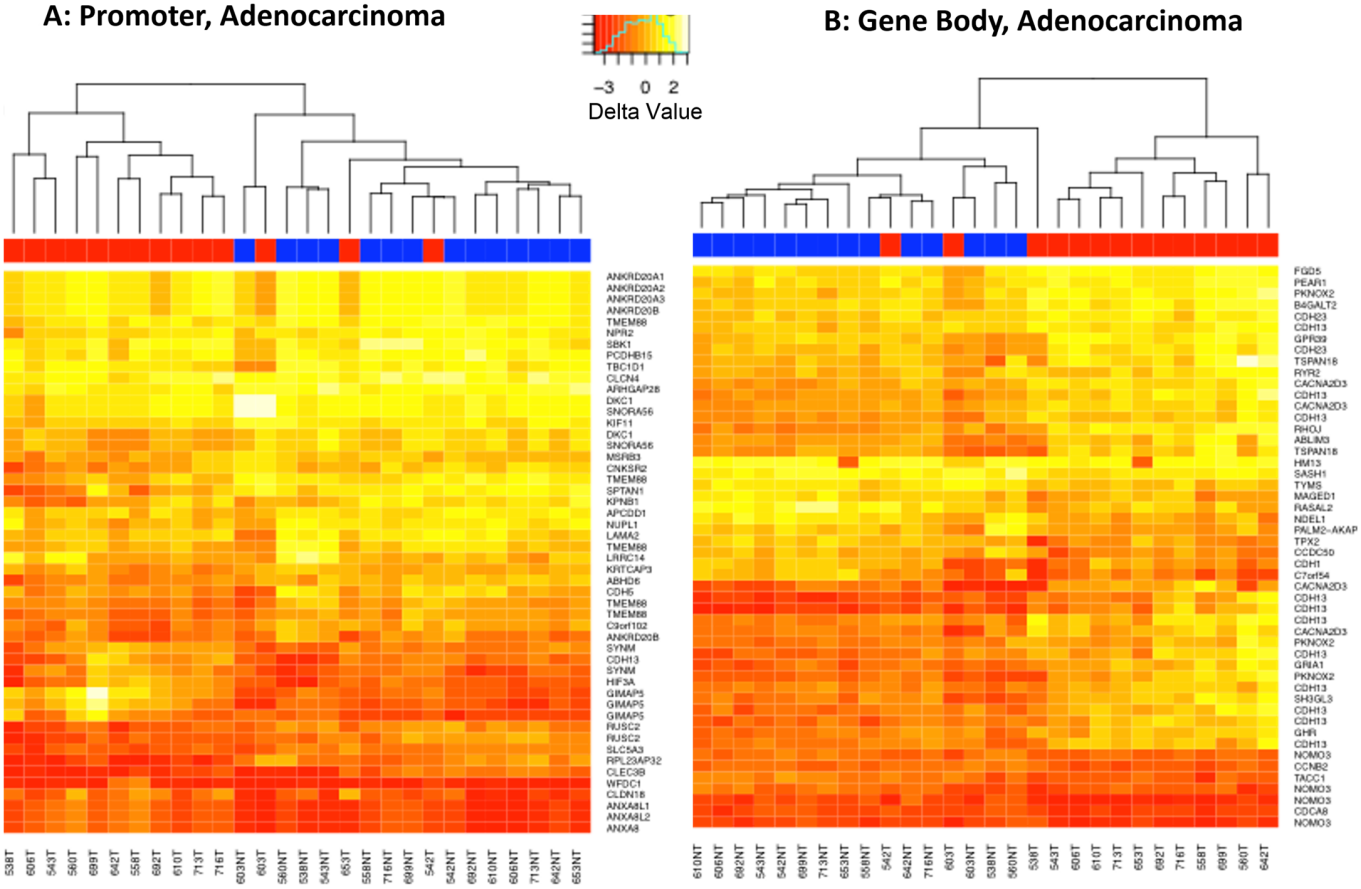
Heat Map of the Top 50 DM Loci within Adenocarcinomas. (A) Promoter regions; and (B) Gene body regions. Several genes show differential methylation (DM) at more than one locus and appear multiple times in the heatmap. Blue = Non Tumor, Red = Tumor.

The top 50 most DM loci (FDR adjusted p<0.05, ranked by magnitude of delta), reflect separation of T and NT in most of the paired samples, except in the samples 603T, 653T and 542T. Several loci within the same gene show DM, resulting in the recurrence of gene names in the heat maps. Those multiple loci within a gene (e.g. PR: *TMEM88*, *GIMAP6*, *RUSC2*, others; GB: *CDH13*, *CACNA203*, *NOMO3*, others) tended to be concordant, albeit imperfectly, with the direction of DM (hyper- vs hypomethylation).

### CpG methylation validation

The methylation states of three representative DM CCGG loci chosen on the basis of DM magnitude (one in the promoter of DARS and two in the promoter of RGS3) were quantitatively determined by the high resolution Sequenom MassARRAY^®^ method, and compared with the results from the HELP microarray-based assay using Spearman rank order correlation software [27]. The correlation (rho) was 0.72 (p = 0.0006), indicating that the results of HELP assay significantly correlated with the reference results of the Sequenom MassARRAY^®^ reference assay (S1 Fig)

### Identification of discriminatory classifiers

The average accuracy for top 100 or top 25 DM loci tumor versus non-tumor classification models, all NSCLC histologies in aggregate, was 87% and 90% respectively. On the adenocarcinoma data subset, the average accuracy for top 100 and top 25 DM loci was 80% and 79% respectivelyIn general, the classification models tend to be more specific than sensitive. (S7 Table). Two loci (*LOXL4* and *LINC00841*) were repeatedly selected within the top DM loci during the classification process.

### Methylation x Expression Merge

DNA loci were integrated with previously generated mRNA transcriptome microarray data among the 21 T-NT pairs where both datasets were available. (S2 Fig). This analysis yielded n = 433,666 DM loci in all compartments (Table 2). For example, pooling all histologies, we identified n = 75 loci that showed hypermethylation in PR regions and concurrent down-regulation of mRNA expression by microarray. There were n = 219 loci within GB regions that showed concurrent hypermethylation and up-regulation of expression.

**Table 2:** Merge of Differential Methylation and Differential Expression (All Histologies– 21 pairs). All Histologies (21 pairs).

**Assay**	**Total # loci**	**FDR p < 0.05**
HELP	1135549	433666
Gene Expression	18208	7957
**Regions**	**Correlation**	**# of loci**
Promoter	Hypermethylated and Downregulated	75
	Hypomethylated and Upregulated	38
	other	3113
Genebody	Hypermethylated and Upregulated	219
	Hypomethylated and Downregulated	3753
	Other	71542
	Total	78740

**Fig 4:**
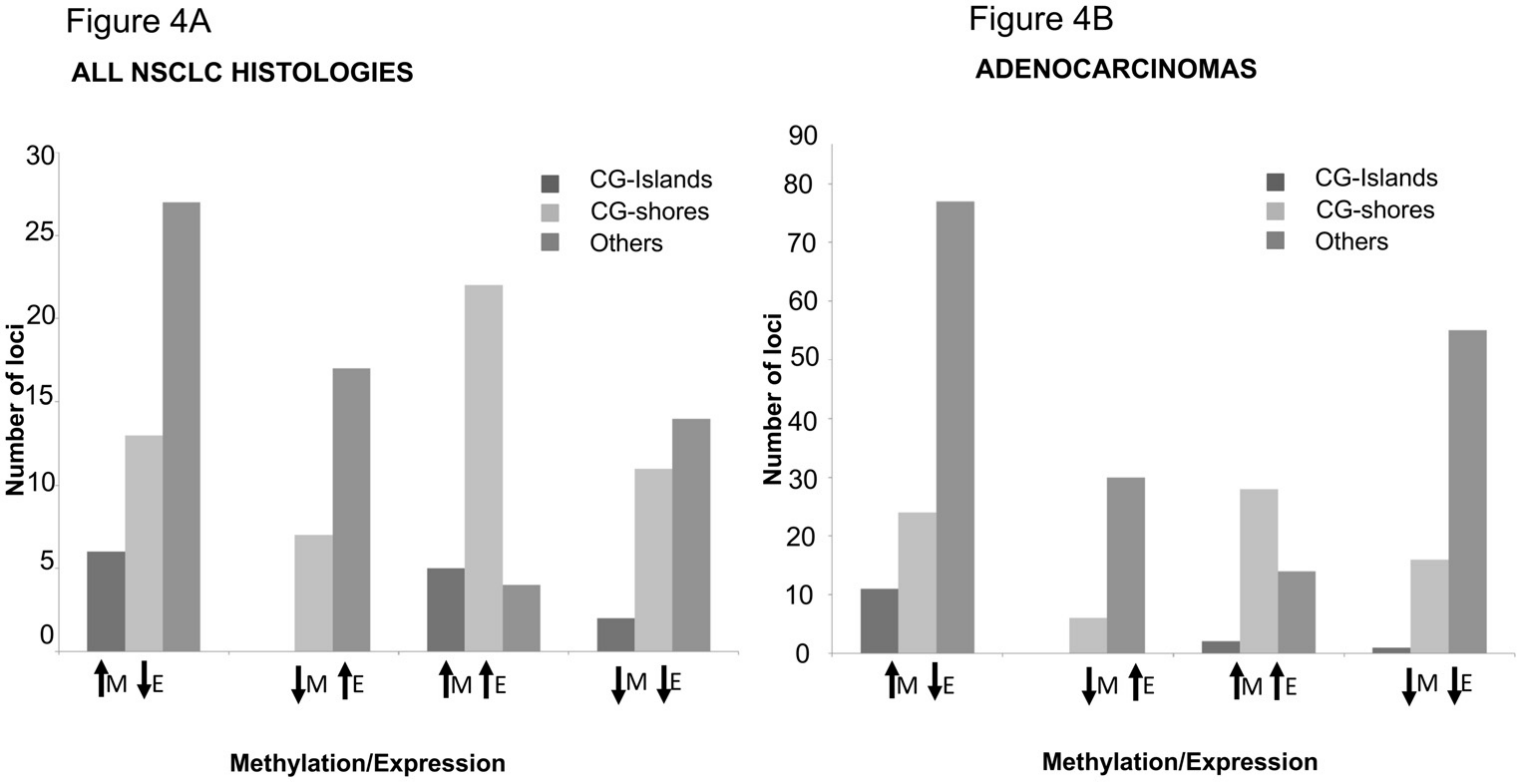
Methylation vs Expression in Promoter regions. Analysis of DM loci within promoter regions and their overlap with differential gene expression. (Left panel A) All 21 pairs (all NSCLC histologies), overall differences do vary by PR genomic location (CGI, CGS, other), ChiSquare p = 3.32E-4. *(Right panel B)* Within the set of adenocarcinomas overall differences do vary by PR genomic location (CGI, CGS, other), ChiSquare p = 1.10E-7. Majority of DM promoter loci are associated with hypermethylation when the DM loci are within CG islands. This effect is more pronounced among adenocarcinomas, where the DM loci in CG islands are mostly associated with downregulation of the gene. KEY: “M” = methylation, “E” = expression. Upward arrow indicates increase and downward arrow indicates decrease.

The promoter-specific subcompartment distribution (CGI, CGS, other) of canonical DMxGE relationships (*e*.*g*. promoter hypermethylation: gene down-regulation) *versus* non-canonical relationships (*e*.*g*. promoter hypermethylation: gene up-regulation) is displayed in Fig 4. Overall, within PR regions, CGI patterns tended to follow canonical DM:GE patterns (first bar of each of the leftmost two bargraph triplets within a panel) somewhat less frequently than the other promoter compartments. Notable is that non-canonical DM:DE relationships were approximately equal in overall frequency to canonical relationships, as assessed by this analysis. For all 21 pairs (all NSCLC histologies, Fig 4A), overall distribution of DMxDE differences do vary by PR genomic location (CGI, CGS, other), ChiSquare p = 3.32E-4. Similarly, within the set of adenocarcinomas (Fig 4B), overall DMxDE differences do vary by PR genomic location (CGI, CGS, other), ChiSquare p = 1.10E-7. The majority of PR DM loci are associated with hypermethylation when the DM loci are within CG islands. This effect is notable among the adenocarcinomas subset as well, where the DM hypermethylated loci in CG islands are mostly associated with downregulation of the gene.

The number of loci obtained from expression-methylation overlay is displayed in 3D coordinates in panel A (S3 Fig). Genomic coordinates were also displayed by circos plots; an example of chromosome 3 is displayed in panel B (S3 Fig). These panels denote the overall patterns of gene body and promoter methylation and accompanying gene expression changes. The overlap between DM and GE for GB was more frequent than PR (in part due to relative over-representation of the GB *versus* PR regions on the HELP microarray (Table 1). The canonical pattern most often seen in GB was hypomethylation and down-regulation (S3 Fig).

Examples of the quantitative relationship of DM to GE in promoters and gene bodies are displayed in selected scatter plots (S4 Fig). Most genes that were qualitatively canonical for the DM⬄GE relationship showed an ambiguous quantitative relationship (not displayed); those genes that are selected for display do exemplify a canonical relationship.

The top eight differentially expressed (DE) genes associated with promoter DM loci (S3 Table: *FILIP*, *HBEF*, *TMEM88*, *VWR*, *CASP12*, *NQO1*, *CST*, *XAGE1D*) underwent a quantitative verification of microarray-based GE expression changes, using qRT-PCR scaled to *GAPDH* internal housekeeper. S5 Fig displays these results, showing general concordance of direction of GE between the two platforms (microarray and qRT-PCR; *r* ^*2*^ = 0.9367815, p< 0.0007), albeit with a compressed dynamic range of the microarray data, as is typical in the literature [28].

### Individual genes revealed by DM x GE Merge

*All NSCLC histologies*: Overlay of DM x DE overlay (S3 Table) yielded additional genomic DM loci with canonical expression patterns (*e*.*g*. PR hypermethylation:mRNA downregulated and PR hypomethylation:mRNA upregulated; GB hypermethylated:mRNA upregulated and hypomethylated:mRNA downregulated) [PR n = 113; GB n = 3972] (Table 2). Notable hypermethylated PR loci with reciprocal decreased expression GE were *HBEGF*, *DPT*, *AGER*, *SPARCL1*, *PTPRM*, *ARHGEF6*, *TMEM88*, *SEMA6A*. Those PR hypomethylated loci with increased GE were *NQO1*, *CST1*, *TNS4*, *FUT2*, *MELK*, *FAM83A*, *MMP9*, *and SLCO1B3*. Those GB loci with concordant methylation and expression included: hypermethylated/increased GE: *FERMT1*, *SLC7A5*, *FAP*, *KRT15*, *ETV4*, *TFAP2a TPX2*, *FOXM1*; hypomethylated/decreased GE: *AGBL1*, *RHOJ*, *LDB2*, *GHR*, *ITGA8*, *ABCB1*, *SEMA5A*, *GPM6A*.

Within the category of adenocarcinomas alone, we merged the results of DM with DE, and discovered several loci with differential methylation in promoters or gene bodies and cognate gene expression changes. Hypermethylated PR loci with GE downregulation include *RPL23AP32*, *CTNNAL1*, *HBEGF*, *TMEM88 and CASP12*. Loci showing PR hypomethylation and upregulation include *NQO1*, *CST1*, *XAGE1D*, *IGKC and AIM2*. GB loci showing hypermethylation and upregulation include *FAP*, *NLN*, *TPX2*, *and KIF26B* and others. GB loci showing hypomethylation and downregulation include *AGBL1*, *RHOJ*, *LDB2*, *GHR*, ITGA8* and others (S3 Table)

### Methylation-Expression relationship in CG-islands and CG-shores

We queried the association between DM and DE for DM loci located within promoter CG islands (CGI) and CG shores (CGS; defined as 2 kb upstream of a CG island; Fig 4; S5 Table). We observed only a small percentage of loci (3–11%) that exhibited DM within CGI or CGS associated with the expected change in gene-expression of the nearest gene. These include genes such as *TMEM88*, *S1P1R*, *FZD4*, *GIPC2*, *DNAJB4*, *ADAMTS1* (hypermethylated in CGI and CGS and downregulated) and BUB1 (hypomethylated and upregulated).

### Pathway analyses

A tabular summary of IPA analyses is offered in S6 Table. All DM loci (Bejamini-Hochberg adj p < 0.05) corresponding to eight categories (based on genomic compartment, histology and with/without gene expression merge) were separately analyzed using IPA, to identify gene networks enriched within the sets of DM loci. In all the eight cases, “Cancer” was the top disease associated with the input data set, although the constituent genes were different. Canonical pathways from Ingenuity’s knowledge base that were found to be enriched within the gene sets with adj. p < 0.05 are reported. Three of the networks (All NSCLC histologies, DM only, GB; Adenocarcinomas DM only, GB; and Adenocarcinomas DM+DE both, PR) among the eight categories were found to have a statistically significant association with a canonical pathway with adj. p < 0.05, and are further outlined below.

#### IPA Cancer-related Networks depictions

Networks analysis of those significant networks tabulated in S6 Table are summarized in S6 Fig. The displays show that several genes that play an important role in cancer-related pathways are differentially methylated in T relative to NT in various categories, and highlights some genes that are not known to do so. For example, pooling all NSCLC histologies, S6A Fig shows the cancer-related network derived from IPA of all DM loci (adj. p<0.05) within GB across all the 24 T/NT pairs. Genes such as *EZH2*, *CDH1*, *CDKN2A* and *DNMT3A/3B* are found at central points in this network. *EZH2* (hypomethylated) is a member of the Polycomb-group family and plays an important role in cell proliferation, growth, cell cycle progression, transcriptional repression and invasion. *DNMT3A*/3B (hypermethylated) encodes a DNA methyl-transferase that is purported to carry out de novo methylation and has an important role in transcriptional repressional signaling. *CDH1* (hypermethylated) encodes E-Cadherin, a known surface adhesion molecule downregulated in cancers. CDKN2A (hypermethylated) is an inhibitor of CDK4 kinase and is a significant tumor suppressor gene, known to be mutated or deleted in different cancers. *ZEB1*, GB hypomethylated, is also highlighted as a central node in this cancer-related network. It encodes a zinc finger transcription factor (also known as *TCF8*) which is known to be an inducer of epithelial-mesenchymal transition in NSCLC [29].

Within the category of adenocarcinomas specifically (16 pairs S6B Fig shows the cancer-related gene network identified from the most significant (adjusted p<0.05) DM loci (within GB). A central hub of this network is the gene *AR* (androgen receptor) which is found to be GB hypomethylated in tumors. AR is a transcription factor activated by the steroid hormone androgen. It plays an important role in cell-growth, proliferation, cell-death and invasion. Because of an apparent centrality in this particular DM network, we further explored the DM methylation pattern of AR as it relates to gender. We observed that the two relevant DM fragments (within GB) were notable for hypermethylation in GB in males compared to females in NT tissue uniquely (t-test FDR, fragment 1 = 0.041, fragment 2 = 3.76E-5). Supervillin *(SVIL)*, is also a gene at a hub of this network (S6B Fig), and is GB hypomethylated in tumors. *SVIL* is a peripheral membrane protein that regulates cell motility, spreading and is known to enhance cell survival by interacting with the tumour suppressor gene p53 and its downstream targets [30].

Adenocarcinoma-specific promoter DM x DE overlay did highlight familiar (*SMAD6*, *tP53*, *CTNNB1*, *NQO1)* as well as unfamiliar lung cancer IPA nodes (S6C Fig). The cancer-related network derived from this analysis consisted of a single hypomethylated gene promoter (NQO1) at the node of a cluster interacting with *TP53*, *HSP70 and NPM1*. Several hypermethylated gene promoters including *HBEGF*, *SMAD6*, *PTPN13*, *CDH5* and *SFTPC* were found at the periphery of the network. This network is comprised of several genes that are not identified as DM from our study, but do form a part of the network by virtue of their previously published interactions with other DM loci, as depicted in open/white shapes.

### Current vs Former smokers

The sample set of 24 subjects consisted of 11 former smokers and 10 current smokers. We investigated the presence of differentially methylated loci based on smoking status. At adjusted p<0.05 level, no loci were found to be DM between current and former smokers.

## Discussion

We report a methylome comparison survey for a set of NSCLC tumors *versus* the paired non-tumor tissue in surgical resection samples in order to identify genome-wide methylation signatures in lung cancer, and filter them for those germane to gene expression alterations from the same samples. The goal is informing diagnostic biomarker work already underway in the laboratory, [31] and target identification for future development in diagnostic and preventive/therapeutic trials [32,33].

Using the HELP assay, we were able to query 1.2 million discontinuous CCGG loci (~1% of the methylome) in a manner representative of all three genomic (PR, GB, IG) regions. Using an FDR adjusted p<0.05 as the cutoff, 452,754 loci across all regions and histologies show statistically significant differential methylation in tumors. The distribution of these DM sites was notably more concentrated in gene bodies than in promoter regions, even considering regional representation variations on the detector microarray, as supported by a permutation test.

Studies thus far have typically focused on promoter methylation in lung cancer, utilizing promoter-focused custom microarrays [10,15,34] or bead arrays [23,35] and thus often query only for pre-selected genomic regions and loci. This is one of the few studies to date that more agnostically examines genome-wide methylation across all regions of the lung cancer genome in multiple samples. The regions of the genome assayed by the HELP assay are dependent upon the occurrences of CCGG sites within the genome, and not by any prior functional or compartment-wise classification of the loci. The HELP assay is less promoter biased (GB and IG regions are represented 3.5x times promoter regions), thereby allowing for the discovery of novel events associated with methylation in other genomic regions in tumor samples [36]. However, the HELP assay misses non-CCGG embedded CpG sites as well as those CCGGs that would define a size range outside the target fragment size range (200-2000bp). HELP (unlike Infinium HM arrays) is not focused on detecting contiguous CpGs of pre-defined gene promoters. While the magnitude of the overall DM differences between tumors and nontumor lung tissue at a given locus tended to be small (generally < 2-fold), reassuring is that the validation of pre-selected DM loci compared favorably with the quantitative reference technique (Sequenom MassARRAY^®^).

Upon examining the gene lists of top DM loci discovered from among other published genome-wide studies in lung cancer to those reported in our study, we found that, as expected, the degree of overlap between our studies and others is modest, possibly indicative of the differences in the HELP platform (which detects fragments bounded by individual CpG sites, but not additional fragment-internal CpG sites), and the target regions queried (which in HELP are equally distributed among PR, GB, and IG regions of the genome. Additionally, we note that the extent of overlap across studies that used the same microarray-based methylation(for e.g. Infinium array) ([23,24,35] was also not large. This could stem from various subject and sample heterogeneity factors, and criteria used to rank DM loci. For example, Sandoval *et al* [35] identified a *HOXA7* region amongst the top most variable CpG promoters, whereas the same locus is not reported in the TCGA study [24] that utilizes the same platform. On the other hand, both these studies report differential methylation of the *HOXA9* locus. Both of these loci do not figure in the lists of top DM loci from our study.

Overall findings from this study include that: there are many individual DM loci/genes, particularly in gene bodies (S2 and S3 Tables); there are many non-canonical DMxDE relationships; and genelists and network relationships include both previously recognized and myriad previously unrecognized loci. As previously recognized, the genome is overall more hypomethylated, but also displays promoter hypermethylation in cancer versus paired non-cancer tissues, as was true from early genome-wide studies of differential methylation in lung cancer [2,21–23]. However, many features clearly differ; for example, we observed no significant overlap with CIMP—based classifications [24,25,37], this was possibly due to the difference in the assays used to determine DM (ours more comprehensive, and included many GB abd IG regions), as well as the limited sample size, and therefore power, of this study.

We uniquely report here the list of genes showing gene-body (GB) methylation alterations in a group of NSCLCs. This finding is of interest as gene body methylation is an understudied phenomenon and the biological effect is not fully understood; gene expression effects from promoter methylation alterations are much better understood [3–5,38]. We also found genome-wide hypomethylation in NSCLC tumors is especially pronounced in the intergenic regions, not previously well explored, and representing the largest proportion of the genome overall.

Differential methylation may have functional impact based largely on sequence and structural chromatin context. That is, while cytosine 5-methylation may silence genes or activate genes, depending on precise position and pattern in the promoter, in intergenic and intronic regions, it may have as much to do with gene splicing and effects at a distance, as with expression of the most proximate, neighboring genes [39,40].

The lists of DM loci in promoters (PR) and gene bodies (GB) revealed many sites that were different from the typical list of methylation silenced “players” in NSCLC, derived from assays that are often *a priori* selected, candidate-gene focused. Clearly, overlap of some of these DM-detected genes with those in the recent literature was apparent in promoters with prior genome surveys [PR: *SLC27A6*, *SIRPB1*; GB: *CNTNAP5*, *CDH13*]. However, many “hits” in this study with no readily apparent representation in the lung cancer methylome-related literature were found, [e.g., PR: *DARS*, *CLDN18*, *APIP;* GB: *ARHGEF12 PRKCE*], and are likely worthy of pursuit. The potential relevance of some of the unique DM genes identified here is described in Table 4i [41–46].

As for the general magnitude of DM in tumors, we observed that hypermethylation changes were generally higher in magnitude across all genomic compartments (PR, GB, IG), predominantly greater than 50% increased, as compared to the magnitude of hypomethylation changes That is, hypermethylation in tumors was predominantly between 1.5–2-fold, albeit notably more common within CGI regions than was hypomethylation.

Given that our study employed homogenized, non-microdissected tissue samples by necessity of the platforms available at study commencement, the mixed cell populations could obscure changes in individual cell types from being identified. Also, the now-appreciated relevance of more local effects of higher resolution patterns of CpG methylation [4,16] within each CCGG-defined fragment could not be assessed with this HELP platform, such that weak or even powerful effects from smaller fragments or motif fine details could not be ascertained at this resolution, and requires high resolution sequencing based follow-up, examples of which are displayed in this report. Notable is that DNA methylation, despite some inter-locus concordance observed here (e.g. *CDH13*, others), is not “linked” to anywhere near the degree of linkage disequilibrium of native germline nucleotide sequence itself, so that inferences of DNA methylation status at even modest (1kb) distances carry much uncertainty [4].

To further characterize the functional effects of PR and GB methylation, we then analyzed differential mRNA expression data generated from the same donated lung resection specimen and examined the overlay with differentially methylated loci. The idea was to use gene expression as a filter for DM changes, to ascertain those DM sites more likely to have functional consequence. We used a simple approach to cross-platform integromics. Using a t-test comparison, after correcting for false discovery rate, we identified among 37,056 DM sites of which only 3,216 were canonically related (DM loci within a 2 kb vicinity of a qualitatively differentially expressed gene, in the expected direction). The majority of these DMxDE canonical loci were in the GB. Among adenocarcinomas, only a small fraction of these DMxDE loci (8.7%) show the expected canonical association between methylation and gene-expression (PR hyper/hypo-methylation: down/up-regulation, respectively, n = 100; GB hyper/hypo-methylation and up/down-regulation, respectively, n = 3136) (Table 3). This could imply that for the vast majority of statistically significant DM loci within a 2 kb vicinity of differentially expressed genes in tumor, the DMxDE “co-occurrence” is coincidental, with no functional implication. Or alternately, this implies that the DMxDE relationship relies instead on more high resolution detail at single CpG site resolution, rather than estimates of overall fragment methylation [4], as is inherent to the HELP assay. Of course, many competing non-CpG methylation inputs to gene expression are also likely.

**Table 3:** Merge of Differential Methylation and Differential Expression (Adenocarcinomas only).

**Assay**	**Total # Loci**	**FDR p < 0.05**
HELP	1135549	225350
Gene Expression	18208	6378
**Regions**	**Correlation**	**# of loci**
Promoter	Hypermethylated and Downregulated	64
	Hypomethylated and Upregulated	16
	other	1239
Genebody	Hypermethylated and Upregulated	138
	Hypomethylated and Downregulated	2998
	Other	32601
	Total	37056

With the current dataset, the genes so discovered in this functional (DMxDE) subset, where methylation does relate to expression, could be appropriate candidates to prioritize to further understand the functional impact of DNA methylation on gene expression, and its role in lung tumor biology. These merged DMxDE datasets suggest some known or previously reported/suspected cancer genes [PR: *SPARCL1*, *NQO1*, *CST1*, *MELK1*, *DPT*, *FAM83A*, *MMP9*; GB: *FOXM1*, *TFAP2*, *GREM1*, *ITGA8*, *GRIA1*, *SLIT3*] as well as many previously unreported genes/loci. The known relevance of some of the above mentioned genes, identified through the DMxDE screen is summarized in Table 4 [47–52]. While provocative in this ‘omics level screen, each of these putative deregulated candidates requires technical as well as biological validation to verify that the DMxDE relationship does indeed exist. We undertook technical validation of gene-expression levels of eight genes identified through the DMxDE analysis: *NQO1*, *CST1*, *XAGE1D* (PR: hypomethylated and up-regulated) and *FILIP1*, *HBEGF*, *TMEM88*, *VWR and CASP12* (PR: hypermethylated and down-regulated) and confirmed the qualitative (up/down) gene-expression regulation, as assessed by qRT-PCR. The magnitude of fold-changes observed by qRT-PCR were larger than those observed by the genome-wide microarray for most genes; that may be explained by the differences in inherent normalization procedures for the two techniques, as well as the ability for qRT-PCR to span a larger dynamic range of mRNA levels [28].

**Table 4:** Relevance of some novel DM and DM+DE genes in Lung cancer.

Gene name	Function	Change identified	Ref
*DARS*	Aspartyl-tRNA synthetase, a member of a multienzyme complex that mediates the attachment of amino acids to their cognate tRNAs. It is also known to function in inducing cell proliferation in its non-canonical role	PR hypermethylated	**[41]**
*CLDN18*	Claudin 18 is an integral membrane protein identified as an early stage marker of pancreatic cancer. Claudins are involved in the regulation of epithelial-cell barrier function and polarity. CLDN18 was previously identified as a potential therapy target in NSCLC by virtue of its overexpression	PR hypomethylated	**[42,43]**
*APIP*	APAF1-interacting protein which functions in the methionine salvage pathway and plays a role in apoptosis. It has been reported to be downregulated in NSCLC at the mRNA and protein level [44](44) and the same study has also shown that other mechanisms in addition to DNA methylation may be involved in its regulation	PR hypermethylated	**[44]**
*PRKCE*	(Protein Kinase C, epsilon) is a member of the serine-threonine protein kinase C (PKC) family with a role in diverse cellular signaling pathways including cell adhesion, motility, migration, cell cycle functions, cancer cell invasion and apoptosis. *PRKCE* overexpression has been associated with tumor aggressiveness, malignant transformation and metastases in a variety of cancers including mammary, prostate and lung cancer.	GB hypermethylated	**[45,46]**
*CST1*	Cystatin SN) (PR hypomethylated and upregulated) is a cysteine protease inhibitor with a role in inflammation and tumorigenesis. It was identified as a target of the Wnt signaling pathway and reported to be overexpressed in endometroid and colorectal cancers. It has also been reported to be overexpressed in NSCLC, and its overexpression is associated with increased risk of recurrence, metastasis and poor survival in NSCLC patients that have undergone surgical resection	PR hypomethylated and expression upregulated	**[47]**
*DPT*	Dermatopontin is a matricellular protein that accelerates collagen fibrinogenesis and may play an important role in wound healing. It is known to be downregulated in oral and hepatocellular carcinoma (HCC). Silencing of *DPT* in HCC has been shown to be mediated by DNA methylation. While *DPT* has been reported to downregulated in the normal bronchial epithelium of smokers relative to non-smokers	PR hypermethylated and expression downregulated	**[48,49]**
*MELK*	MELK is a serine threonine protein kinase is known to be upregulated in lung, colon, breast, and ovarian cancers. It has been identified as a promising drug target and a MELK-inhibitor molecule OTSSP167 has been developed and is currently undergoing Phase III trials	PR hypomethylated and expression upregulated	**[50, 51]**
*FOXM1*	FOXM1 is a transcription factor that regulates the expression of cell-cycle genes. It is regarded as a proto-oncogene and is upregulated in several cancers including NSCLC. In NSCLC, it is required for K-Ras-mediated tumorigenesis by activating NF-κB and JNK pathways	GB hypermethylated and expression upregulated	**[52]**

When examining the gene networks formed from groups of DM genes in various categories using IPA analysis, there were several networks where both known and unknown lung cancer genes/nodes were apparent. For example, the zinc finger transcription factor *ZEB1* (*TCF8*) (GB hypomethylated) was identified within the IPA network generated from GB DM loci, all NSCLC histologies (S6A Fig). While the role of *ZEB1* as an inducer of EMT (epithelial-mesenchymal transition) in NSCLC is well studied [53] and, its regulation by miR-200c has been reported [54], it is yet to be determined if differential gene body methylation observed in this study confers an additional level of gene expression regulation. If indeed GB hypomethylation is found to be tightly associated with *ZEB1* expression, it can potentially serve as a biomarker of erlotinib resistance by virtue of its role in EMT [55].

The refined GB, DM set of genes for adenocarcinoma specifically showed enrichment for cancer-related canonical pathways (BH adjusted p-value = 0.0152). The top network in this subset displayed a centrality of the androgen receptor (*AR*), not generally implicated in lung cancer to date. We noted that AR differed in expression across gender in the non-tumor compartment, but was not gender-specific in the tumor compartment. *SVIL* (supervilin) (GB:hypomethylated) is involved in actin-myosin and cell spreading, a plausible but unexpected finding in lung cancer as well.

A new network discovery pattern was also apparent for the DMxDE merged datasets, even if the DM locus in isolation was not readily apparent in the IPA nodes. For example, examining the refined adenocarcinoma only, PR only, DMxDE network for adenocarcinoma displayed the known cancer-related genes [*TP53*, *Akt*, *NQO1*], but also myriad additional nodes [*SMARCA4*, *ITGB1*, *CTNNB1*, *Hsp70*, *AR*, others], to date of unknown significance. Similarly, *DACH1* (PR hypermethylated in tumor) was one of the IPA-defined nodes identified as a chromatin-binding protein that associates with other transcription factors to govern gene-expression during development. *DACH1* expression has been reported to be reduced in human NSCLC where it was determined to bind tp53 and block lung adenocarcinoma cell growth [56].

Our study was necessarily limited in sample size to accommodate this dense two-platform analysis within available resources, and therefore did not detect DM loci in squamous cell carcinomas within the statistical threshold applied (adjusted p < 0.05) to any significant degree. This was most likely due to the smaller number of squamous cell carcinoma samples (n = 6) available to us for multiplatform analysis at the time/funding of the study. Similarly, the overall small sample size may have precluded the robust identification of statistically significant changes in current vs former smokers. Another possibility, however, is that tobacco smoke-induced methylation changes are persistent, and incompletely reversed by smoking cessation, in both tumor and non-tumor tissue alike, which is compatible with the epidemiology [57].

We were unable to evaluate EGFR/KRAS somatic mutation data for adenocarcinoma subgrouping, as most resections accrued before this was routine somatic mutation clinical testing, and *post-hoc* subject permission was not possible to obtain for many subjects, due to interval subject deaths, and other factors.

In summary, a genome-wide query of DNA methylation in lung cancer was performed, showing significant alterations in gene bodies as well as gene promoters and intergenic regions, including many previously unrecognized loci. An initial integromics overlay of genome-wide DNA methylation with gene expression data yielded many hits and coupled DMxDE nodes, worthy of further validation. One can envision exploration of those potential targets that validate in future observational and experimental studies, for the purposes of risk and diagnostic biomarker development, and for targeted tumor modulation and/or prevention.

## Materials and Methods

(Details available in S1 File)

### Patient recruitment and Sample collection

All subjects were enrolled under, and this study was approved by, the Albert Einstein College of Medicine IRB—protocol (#2007–407). All subjects provided fully informed written consent approved by Albert Einstein College of Medicine IRB. This study was comprised of a total of 30 consenting individuals undergoing lung resectional surgery for clinically suspected non-small cell carcinoma. Patient recruitment was conducted as previously described [58–60]. We surveyed tumor and adjacent non-tumor tissue from the initial 30 donors drawn from our lung cancer tissue repository. Paired tissue samples were collected in the operating room after lobar resection and immediately snap frozen in liquid isopentane within 15 min of surgical resection; and stored in a −180°C liquid nitrogen tissue bank until analyzed. Sections from these snap frozen blocks were examined by a pathologist to confirm tumor presence and composition, to distinguish between adenocarcinomas, squamous cell carcinomas and mixed adenosquamous type.

The assigned clinical surgical pathologist confirmed the diagnosis of lung cancer in all cases, per clinical routine, and classified the samples according to the 1999 WHO histologic classification of lung and pleural tumors, and recent updates [58]. All adenocarcinomas were invasive adenocarcinoma, rather than adenocarcinoma *in situ*, or minimally invasive adenocarcinomas. Additionally, all selected cases were independently re-reviewed by two pathologists (JL, CZ), blinded to prior histologic diagnosis, clinical, and methylome and transcriptome data.

### HELP assay

We used a microarray based version of the HELP assay [26], whereby methylation sensitive (*Hpa*II) *versus* insensitive (*Msp*I) enzyme pair digests the genome, detecting fragments containing paired CCGG sites at the ends of the fragments of 200–2000 bp.

To investigate relative ratios of *Hpa*II and *Msp*I digested products from the same sample, a Nimbelgen whole genome high density tiling microarray was used. This array contained 2.3 million probes corresponding to 1.2 million *Hpa*II sites throughout the human genome. For each sample, *Hpa*II and *Msp*I LM-PCR libraries were labeled with Cy5 and Cy3 dyes respectively and cohybridized to the Nimbelgen array. Array images generated as cel files were pre-processed and then analyzed.

We used custom R-scripts [61] to carry out data preprocessing. Array data were subject to detailed quality control checks (QC) by generating intensity plots. Based on the intensity plots, six pairs were discarded from further analysis due to the presence of non-uniformities and biased intensities. QC-pass array data for 24 pairs were subsequently subjected to normalization and computation of *Hpa*II to *Msp*I ratios using an R pipeline [61].

### Regional validation

In the initial DM-only analysis, differences in T vs NT at the fragment locus level were examined using paired t-tests and an additional test to correct for FDR was also applied [62]. Loci were then ranked by their corresponding p-values, and top-ranked loci (FDR p-value < 0.05) considered for subsequent analyses.

Significance of distribution of DM loci within the various genomic compartments (PR/GB/IG) was tested: 433505 loci (same as the number of statistically DM loci (FDR p<0.05)) were first picked at random. 1000 such iterations were performed to assess compartment-wise distribution of DM loci. These distributions were compared to the actual distribution of DM loci observed to determine the statistical significance of over-representation of DM loci within GB regions.

### Technical validation

Statistically significant DM loci were ranked based on the proximity of a locus to other loci showing the same direction of change in methylation, as well as belonging to the same genomic compartment (PR, GB, IG). This strategy helps assess loco-regional methylation consistency across adjacent CCGG-defined fragments. Top ranking promoter loci thus identified (DARS, RGS3) were further evaluated for validation by Sequenom MassARRAY EpiTYPER^®^ [18].

### Identification of discriminatory of classifiers

The complete (all NSCLC histologies) data set, as well as the set of adenocarcinomas alone, were split (2/3 and 1/3) into training and test data sets respectively. The top 25 or 100 DM loci were selected from within the training sets, and the process was repeated iteratively 10 times. The success of these DM loci to separate T and NT samples within the test data set was evaluated.

### Methylation-Expression Correlation

Paired patient samples were processed with HELP assay or expressionmicroarray using Affymetrix HuGene 1.0 st chips. HELP assay’s p values were adjusted for multiple testing using FDR method with R package multi-test function p.adjust (method = “fdr”). Significance was defined by FDR adjusted p value < 0.05 for both HELP loci selection and microarray gene selection.

We performed paired t tests of tumor vs non-tumor samples for HELP assay and expression microarray data separately. Significant HELP loci were correlated with significantly expressed genes by genome location, if methylation loci were located within 2kb upstream of gene transcription starting site, the loci were classified as in promoter region; if loci located within 2 kb downstream of gene transcription starting and upstream/downstream of ending sites, the loci were classified as in gene body region. For promoter-specific analyses, regions within 2 kb upstream of annotated CG islands were classified as CG-shores.

### Pathway Analysis

To determine pathways and networks associated with DM loci, we conducted Ingenuity IPA^®^ analysis. All DM loci (FDR adjusted p< 0.05) as well as DM loci within the vicinity of DE genes were subject to analysis. Fishers t-test was used to assign statistical significance of the association of a given pathway with the set of DM loci. We used multiple-hypothesis corrected p-values to assign significance to the canonical pathways discovered associated with each category of DM loci.

## Supporting Information

S1 Fig Validation of selected individual DM sites. Two index genes were used, *DARS* and *RGS3* gene. *Left panel* A) UCSC genome-browser screen shots for 3 different T/NT pairs; *DARS* and *RGS3* is displayed. Red indicates Tumor, and blue indicates Non-Tumor. Methylated fragments are represented as quantitative Sequenom MassArray EpiTYPER^®^ measurements shown in a thin vertical bar graph from 0–100% methylation. The CpG locus-specific T-NT differences are subtle. *Right panel* B) For RGS3 gene, a 495 bp DNA fragment upstream of the transcription start site (Chr9:116,262,214–116,262,708) was amplified for MassARRAY EpiTYPER^®^ analysis. The methylation state of one CCGG site was quantitatively analyzed from four pairs of tumor and nontumor tissues. For DARS gene, a 308 bp DNA fragment upstream the transcription start site (Chr2:136,744,845–136,745,152) was amplified for Sequenom MassARRAY EpiTYPER analysis. The methylation state of two CCGG sites was quantitatively analyzed from four pairs of tumor and nontumor tissues. The methylation degree was calculated by methylated CCGG/methylated +unmethylated CCGG (Methylation ratio by rank, Y-axis). For HELP assay, the methylation degree was indicated by delta value from *Hpa*II vs *Msp*I (delta value by rank, X-axis). Spearman Rank Order Correlation software was used for analysis. The correlation (rho) was 0.72 (p = 0.0006).
(PDF)

S2 Fig Strategy for arriving at DM loci associated with DE genes (DMxDE). Statistically significant DM loci (FDR p<0.05) within promoters and gene bodies and DE genes (FDR p<0.05) were chosen. These DM loci were queried for position within 2 kb of a DE gene. Such loci thus associated with FDR p<0.05 are considered to be associated with differential gene expression, and the direction and location of DM and DE were further analyzed (Tables 2 and 3).
(PDF)

S3 Fig Integration of DNA methylation (DM) and gene expression (GE) for 14 lung adenocarcinomas *vs*. paired non-tumor samples. *(Left panel*, *A)* Methylome data were overlaid on mRNA expression data for gene promoters *(left)* and gene bodies *(right*, to demonstrate capacity and feasibility. X-axis is the delta readout of the HELP assay; negative (leftward deflection) by convention is for hypermethylated in the test sample tumor, compared to the comparison sample (far-adjacent non-tumor alveolar tissue). Y axis represents the inverse log_10_ of the false discovery rate (FDR), and z axis is log_2_ fold change (mRNA levels in tumor:non-tumor). The color of the dots depict “coherent” patterns, where expected biological relationships are manifest. For example hypermethylation in a promoter region correlates to decreased expression (green dots), whereas hyper-methylation in a gene body correlates with increased mRNA expression (orange). KEY: Red: gene fold change >2 & delta>0 (T hypomethylated); Green: fold change < -2 & delta < 0 (T hypermethylated); Orange: fold change > 2 & delta < 0; Blue: fold change < -2 & delta > 0.
*(Right panel B)* The circos plot for chromosome 3 is an example of mapping deregulated “hotspots” to chromosomal coordinates, and as internal check, here highlights several well-known tumor suppressor and other known cancer-related genes (*MASP1*, *WNT7a*, *TGFBR2*, *GATA2*). Fragments that are hypomethylated are in green (outer circle), HELP tags that are hypermethylated are shown in blue (middle circle); and expression microarray genes are shown in yellow for down-regulation, and red for up-regulation (inner circle). The longer purple lines that cut through the chromosome marked the correlated promoter region, while the shorter brown lines mark the gene body regions.
(PDF)

S4 Fig Scatter plots of select genes depicting canonical relationships between methylation and expression. (PR: hypermethylated, downregulated or hypomethylated, upregulated; and, GB: hypermethylated, upregulated or hypomethylated, downregulated). Only a small fraction of genes (8%) identified from the significant DMxDE overlay analyses displayed these canonical relationships.
(PDF)

S5 Fig Validation of gene-expression changes by qRT-PCR. Verification was performed in top representative genes that show canonical promoter patterns; PR:hypermethylation and GE downregulation and PR:hypomethylation & GE upregulation. Among DE genes associated with promoter DM loci (S3 Table), these eight genes were selected for qRT-PCR quantitation of gene-expression. All fold changes are depicted for T relative to matched NT; gene-expression values were normalized to GAPDH expression levels. Microarray fold-change values are depicted alongside as a reference. PCR primers and conditions used are described in S4 Table.
(PDF)

S6 Fig IPA network analyses. **S6A Fig Top IPA network generated from DM loci within gene bodies from all 24 pairs.** Previously well-known cancer-related genes such as *EZH2*, *CNR1*, *SUZ2* (GB hypomethylated), and *CDH1*, *DNMT3A/B*, *CNR1* (GB hypermethylated) form major nodes in this network. At the periphery of the network several lung cancer—related genes can be detected such as *SFRP5*, *MUC4*, *PTPRF*. **S6B Fig Top Gene network generated from DM loci within gene bodies in Adenocarcinomas alone (16 pairs).** *AR* (GB hypomethylated in tumors) the androgen receptor gene until now not closely associated with lung cancer forms a central node in this network, and was noted to be more methylated in the GB of normal lung tissue of men than women (not shown here). *SVIL* (GB hypomethylated) is involved in actin-myosin and cell spreading, and, connects with several other gnes involved in cytoskeletal function including *MYO1B* (GB hypermethylated), *TUBA*, *TUBB*, *LMNB* etc. **S6C Fig Cancer-related gene network generated from DM loci within promoters in the vicinity of DE genes, Adenocarcinomas alone (13 pairs).** The cancer-related network derived from this analysis consisted of a single hypomethylated gene promoter (*NQO1*) at the node of a cluster interacting with *TP53*, *HSP70 and NPM1*. Several hypermethylated gene promoters including *HBEGF*, *SMAD6*, *PTPN13*, *CDH5* and *SFTPC* were found at the periphery of the networks. This DMxDE network is comprised of several genes that are not identified as DM from this study, but form a part of the network by virtue of their interactions with other DM loci and are depicted in white shapes.
(PDF)

S1 File Supplementary Materials and Methods. (PDF)

S1 Table Donor Characteristics. (PDF)

S2 Table Top 25 Differentially Methylated Loci. (PDF)

S3 Table Methylation-Expression Overlay. (PDF)

S4 Table Primers used. (PDF)

S5 Table Promoter CGI and CGS distinction in DMxDE analysis. (PDF)

S6 Table Summary of IPA Analyses. (PDF)

S7 Table Discrimination Stability of DM Loci Set. (PDF)
