# Supplementary figures and images for: Genome Wide Methylome Alterations in Lung Cancer
Source: PLoS One. 2015 Dec 18;10(12):e0143826. doi: 10.1371/journal.pone.0143826 (PMC4684329; doi:10.1371/journal.pone.0143826)

# Supplemental Figure S1

S1A Fig.

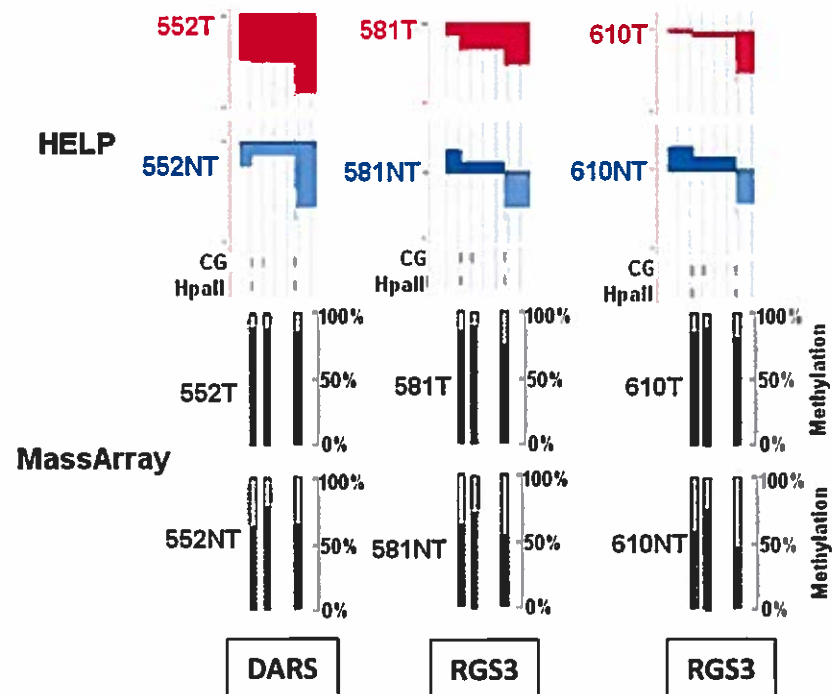

S1B Fig.

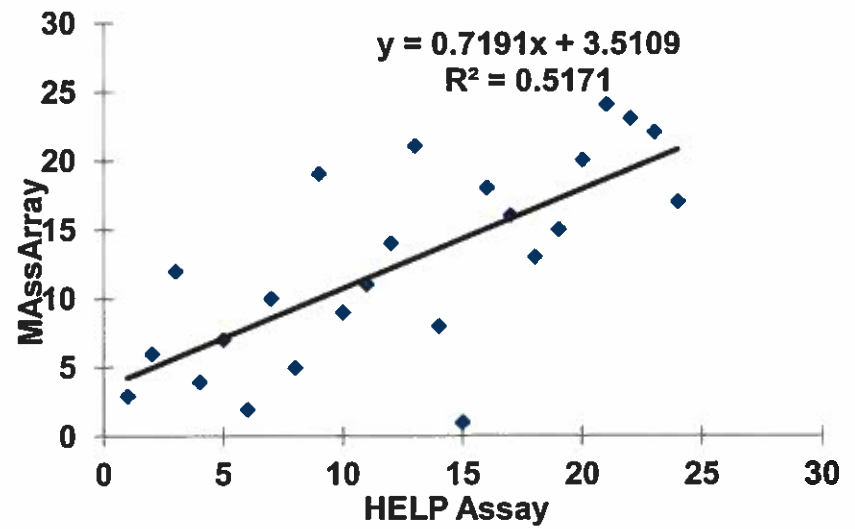

Supplement: S1 Fig — Two index genes were used, DARS and RGS3 gene. Left panel A) UCSC genome-browser screen shots for 3 different T/NT pairs; DARS and RGS3 is displayed. Red indicates Tumor, and blue indicates Non-Tumor. Methylated fragments are represented as quantitative Sequenom MassArray EpiTYPER® measurements shown in a thin vertical bar graph from 0–100% methylation. The CpG locus-specific T-NT differences are subtle. Right panel B) For RGS3 gene, a 495 bp DNA fragment upstream of the transcription start site (Chr9:116,262,214–116,262,708) was amplified for MassARRAY EpiTYPER® analysis. The methylation state of one CCGG site was quantitatively analyzed from four pairs of tumor and nontumor tissues. For DARS gene, a 308 bp DNA fragment upstream the transcription start site (Chr2:136,744,845–136,745,152) was amplified for Sequenom MassARRAY EpiTYPER analysis. The methylation state of two CCGG sites was quantitatively analyzed from four pairs of tumor and nontumor tissues. The methylation degree was calculated by methylated CCGG/methylated +unmethylated CCGG (Methylation ratio by rank, Y-axis). For HELP assay, the methylation degree was indicated by delta value from HpaII vs MspI (delta value by rank, X-axis). Spearman Rank Order Correlation software was used for analysis. The correlation (rho) was 0.72 (p = 0.0006). (PDF) [file pone.0143826.s001.pdf]

## Supplemental Figure S2

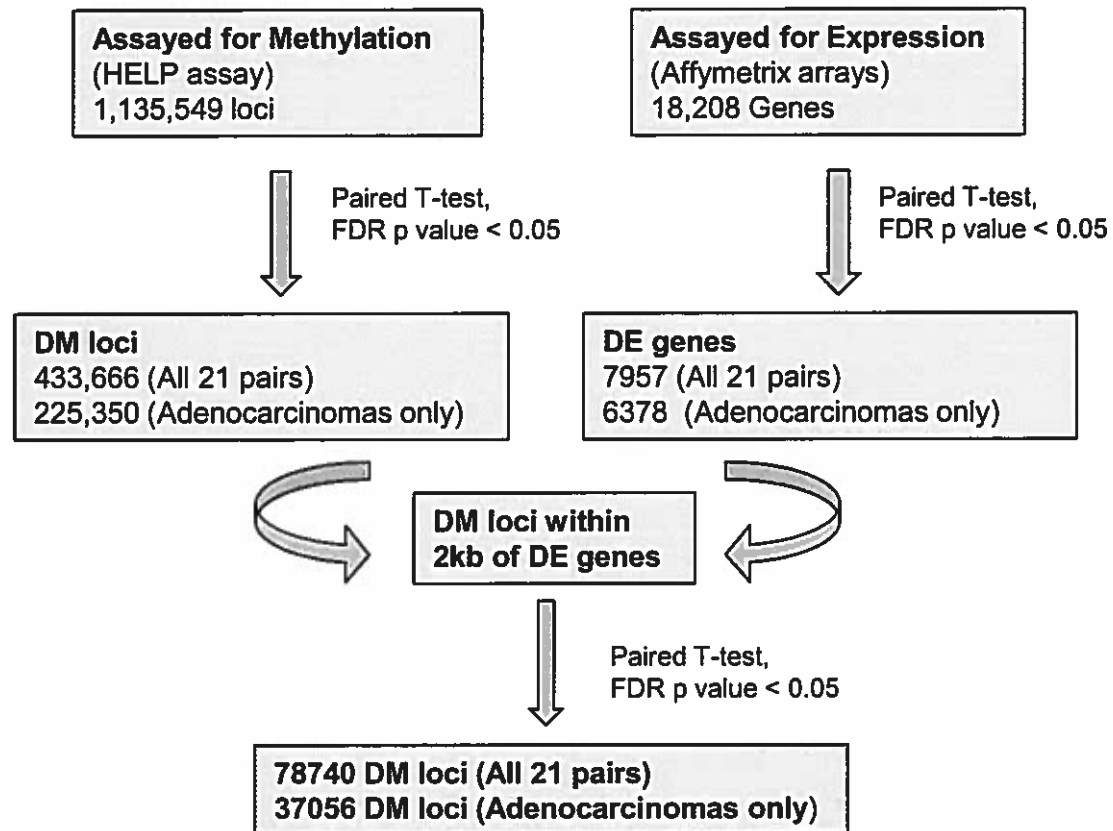

Supplement: S2 Fig — Statistically significant DM loci (FDR p<0.05) within promoters and gene bodies and DE genes (FDR p<0.05) were chosen. These DM loci were queried for position within 2 kb of a DE gene. Such loci thus associated with FDR p<0.05 are considered to be associated with differential gene expression, and the direction and location of DM and DE were further analyzed (Tables 2 and 3). (PDF) [file pone.0143826.s002.pdf]

## Supplemental Figure S3

S3A Fig.

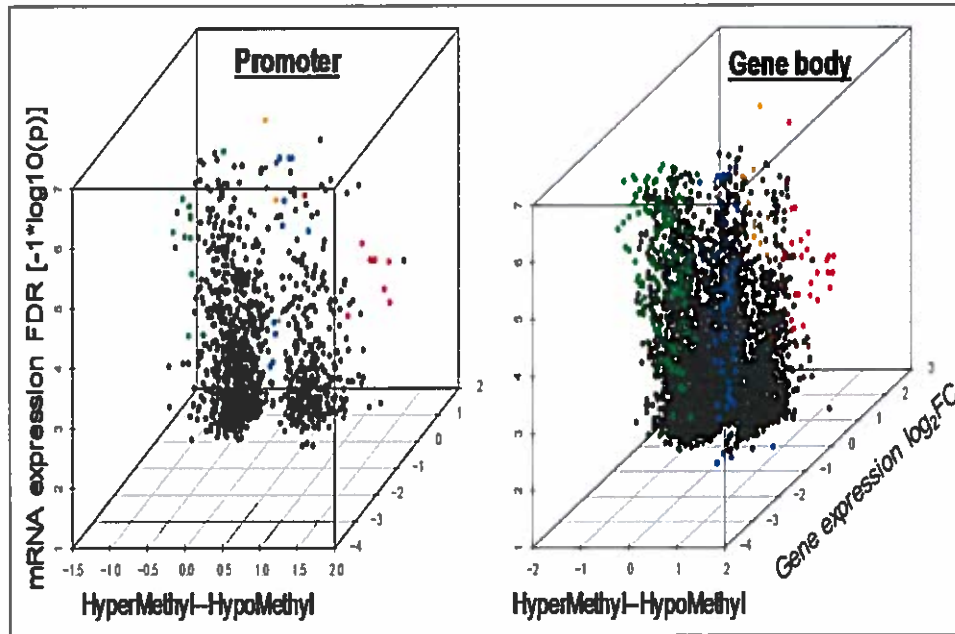

S3B Fig.

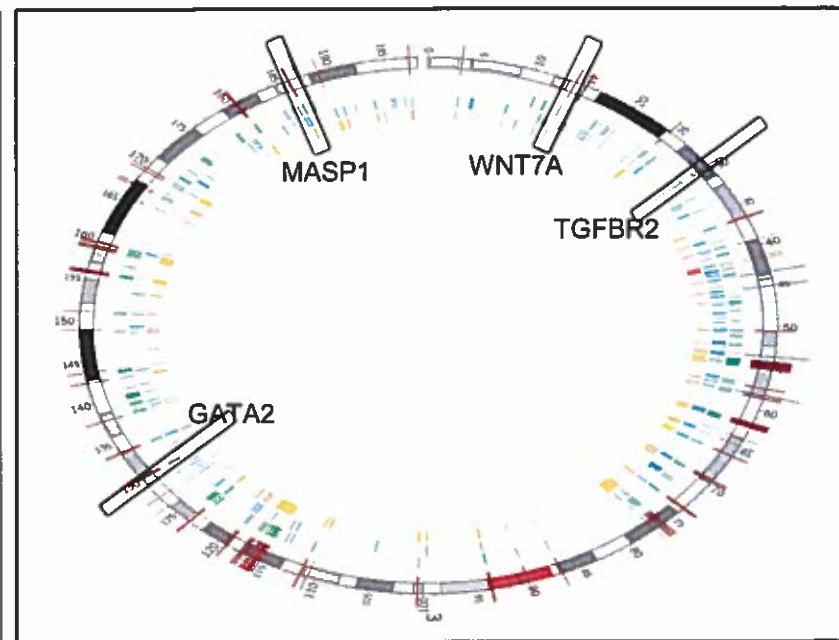

Supplement: S3 Fig — (Left panel, A) Methylome data were overlaid on mRNA expression data for gene promoters (left) and gene bodies (right, to demonstrate capacity and feasibility. X-axis is the delta readout of the HELP assay; negative (leftward deflection) by convention is for hypermethylated in the test sample tumor, compared to the comparison sample (far-adjacent non-tumor alveolar tissue). Y axis represents the inverse log10 of the false discovery rate (FDR), and z axis is log2 fold change (mRNA levels in tumor:non-tumor). The color of the dots depict “coherent” patterns, where expected biological relationships are manifest. For example hypermethylation in a promoter region correlates to decreased expression (green dots), whereas hyper-methylation in a gene body correlates with increased mRNA expression (orange). KEY: Red: gene fold change >2 & delta>0 (T hypomethylated); Green: fold change < -2 & delta < 0 (T hypermethylated); Orange: fold change > 2 & delta < 0; Blue: fold change < -2 & delta > 0. (Right panel B) The circos plot for chromosome 3 is an example of mapping deregulated “hotspots” to chromosomal coordinates, and as internal check, here highlights several well-known tumor suppressor and other known cancer-related genes (MASP1, WNT7a, TGFBR2, GATA2). Fragments that are hypomethylated are in green (outer circle), HELP tags that are hypermethylated are shown in blue (middle circle); and expression microarray genes are shown in yellow for down-regulation, and red for up-regulation (inner circle). The longer purple lines that cut through the chromosome marked the correlated promoter region, while the shorter brown lines mark the gene body regions. (PDF) [file pone.0143826.s003.pdf]

## Suppl. Fig. S4

### PR- HYPERMETHYLATED- DOWNREGULATED

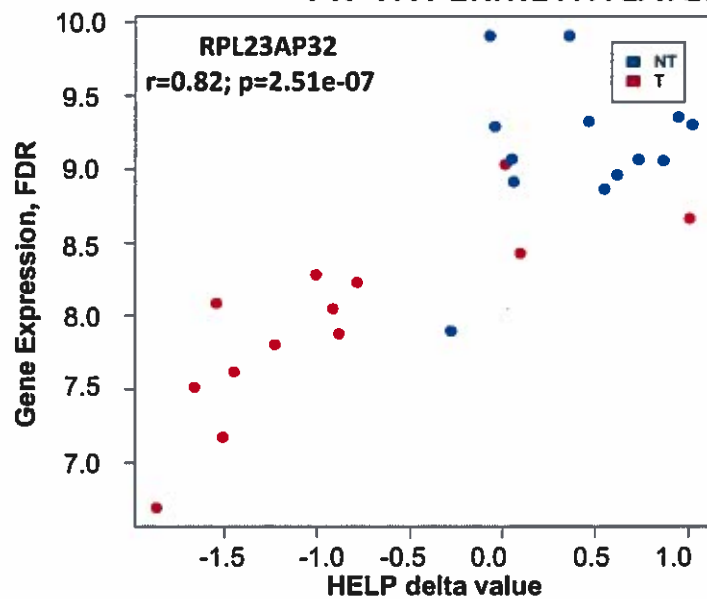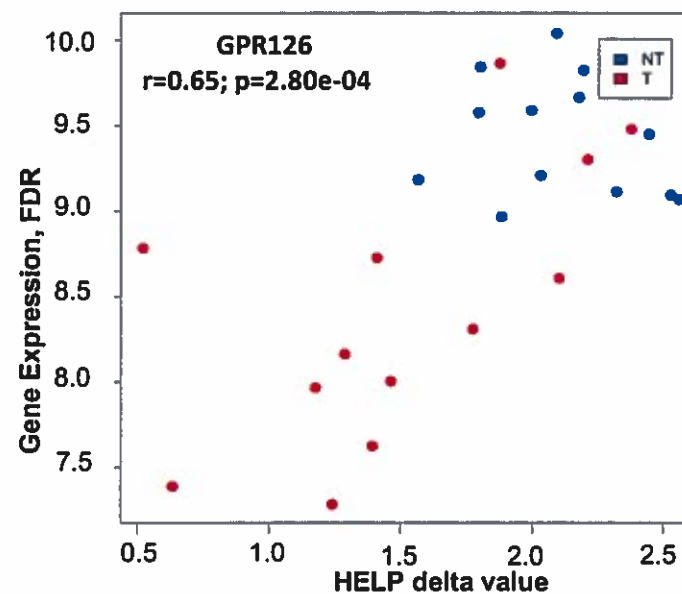

### GB- HYPOMETHYLATED- DOWNREGULATED

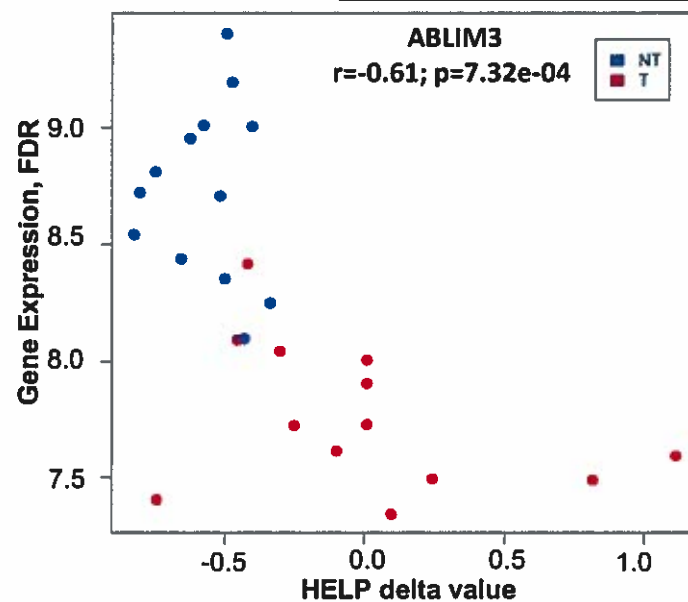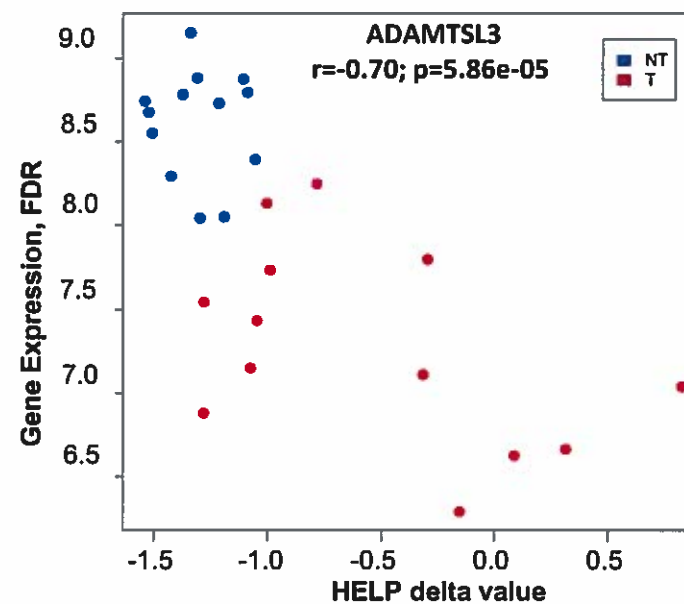

Supplement: S4 Fig — (PR: hypermethylated, downregulated or hypomethylated, upregulated; and, GB: hypermethylated, upregulated or hypomethylated, downregulated). Only a small fraction of genes (8%) identified from the significant DMxDE overlay analyses displayed these canonical relationships. (PDF) [file pone.0143826.s004.pdf]

## Suppl. Fig. S5

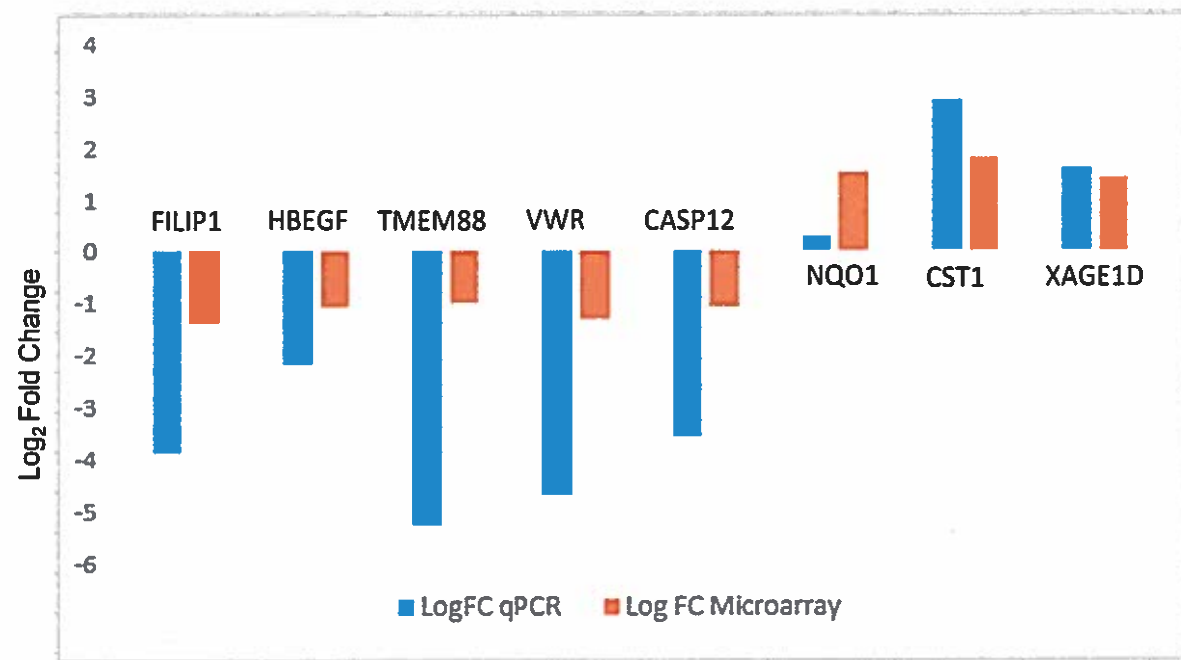

Supplement: S5 Fig — Verification was performed in top representative genes that show canonical promoter patterns; PR:hypermethylation and GE downregulation and PR:hypomethylation & GE upregulation. Among DE genes associated with promoter DM loci (S3 Table), these eight genes were selected for qRT-PCR quantitation of gene-expression. All fold changes are depicted for T relative to matched NT; gene-expression values were normalized to GAPDH expression levels. Microarray fold-change values are depicted alongside as a reference. PCR primers and conditions used are described in S4 Table. (PDF) [file pone.0143826.s005.pdf]
