# Supplementary material for: Genome Wide Methylome Alterations in Lung Cancer
Source: PLoS One. 2015 Dec 18;10(12):e0143826. doi: 10.1371/journal.pone.0143826 (PMC4684329; doi:10.1371/journal.pone.0143826)
Supplement: S6 Fig — S6A Fig Top IPA network generated from DM loci within gene bodies from all 24 pairs. Previously well-known cancer-related genes such as EZH2, CNR1, SUZ2 (GB hypomethylated), and CDH1, DNMT3A/B, CNR1 (GB hypermethylated) form major nodes in this network. At the periphery of the network several lung cancer—related genes can be detected such as SFRP5, MUC4, PTPRF. S6B Fig Top Gene network generated from DM loci within gene bodies in Adenocarcinomas alone (16 pairs). AR (GB hypomethylated in tumors) the androgen receptor gene until now not closely associated with lung cancer forms a central node in this network, and was noted to be more methylated in the GB of normal lung tissue of men than women (not shown here). SVIL (GB hypomethylated) is involved in actin-myosin and cell spreading, and, connects with several other gnes involved in cytoskeletal function including MYO1B (GB hypermethylated), TUBA, TUBB, LMNB etc. S6C Fig Cancer-related gene network generated from DM loci within promoters in the vicinity of DE genes, Adenocarcinomas alone (13 pairs). The cancer-related network derived from this analysis consisted of a single hypomethylated gene promoter (NQO1) at the node of a cluster interacting with TP53, HSP70 and NPM1. Several hypermethylated gene promoters including HBEGF, SMAD6, PTPN13, CDH5 and SFTPC were found at the periphery of the networks. This DMxDE network is comprised of several genes that are not identified as DM from this study, but form a part of the network by virtue of their interactions with other DM loci and are depicted in white shapes. (PDF) [file pone.0143826.s006.pdf]

Supplemental Figure S6

[Legend common to all 3 panels (S6A, S6B, S6C)]

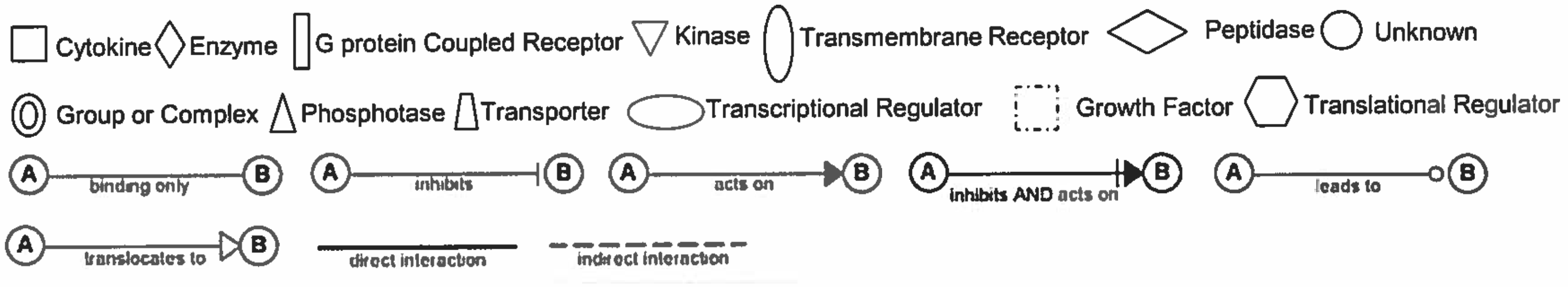

Red = hypomethylated, Green = hypermethylated. White = Not detected as DM but included in the network by virtue of association with at least one DM gene.

S6A

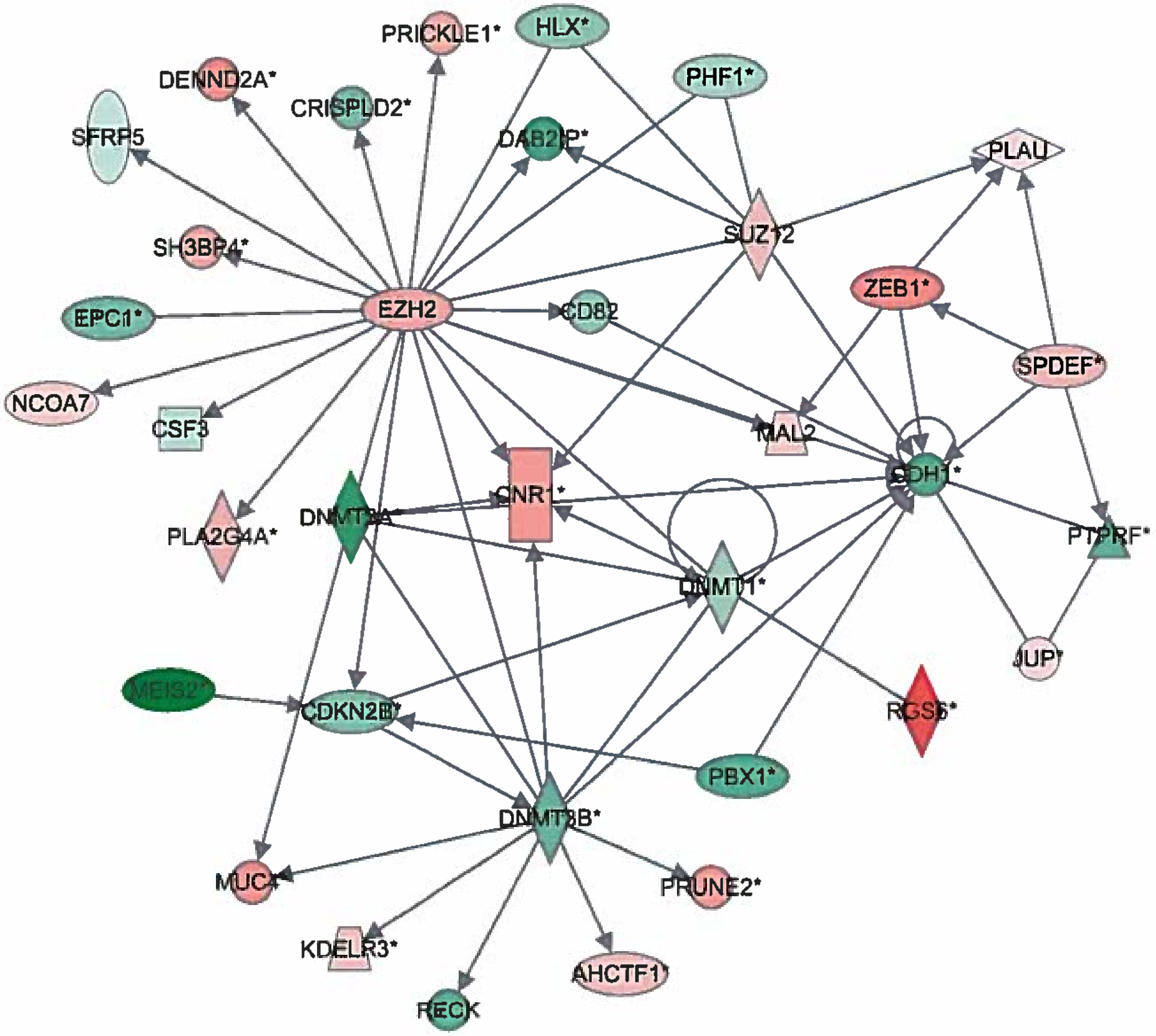

DM LOCI WITHIN GENE BODIES,
ALL NSCLC HISTOLOGIES

S6B

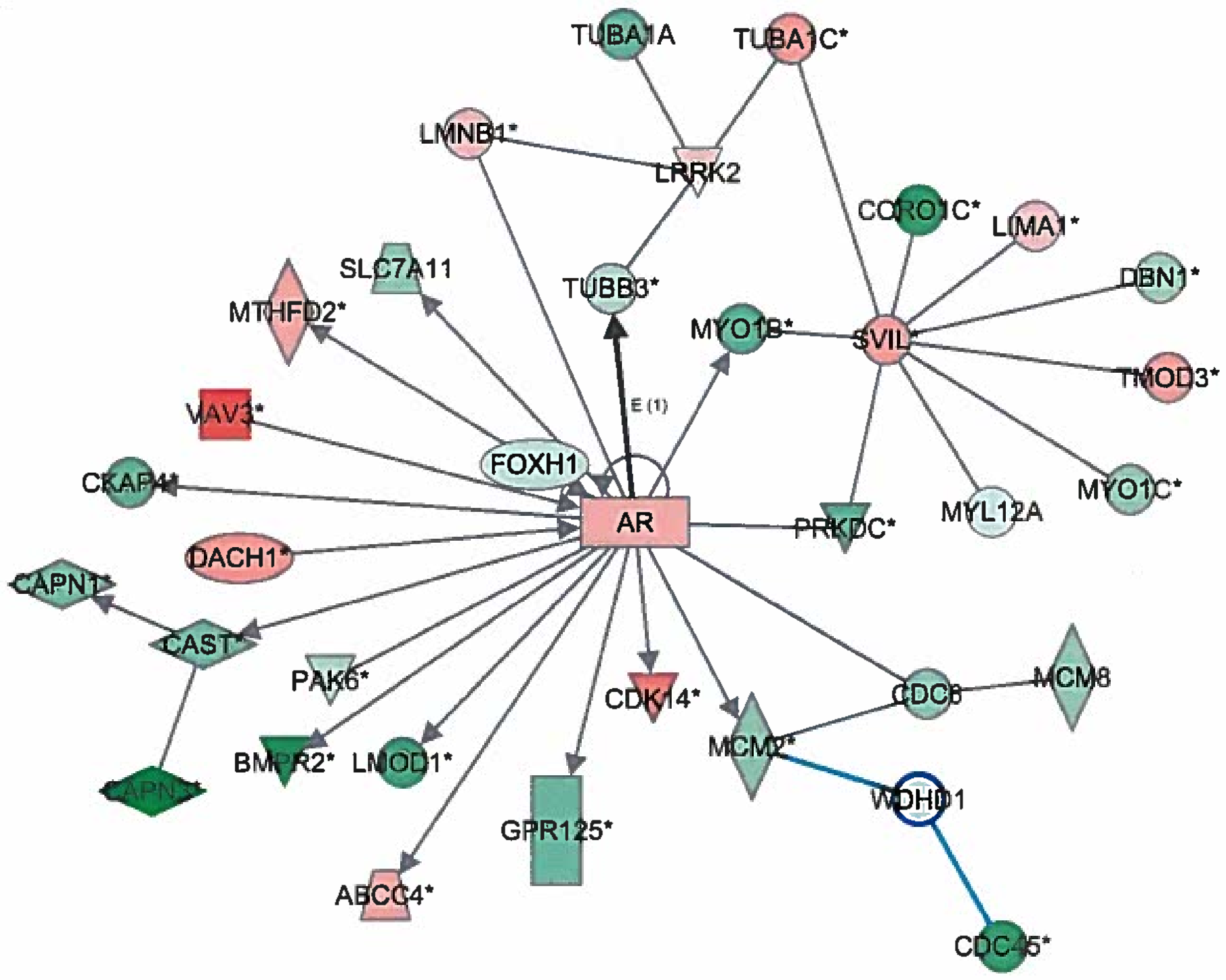

DM LOCI WITHIN GENE BODIES, ADENOCARCINOMAS only

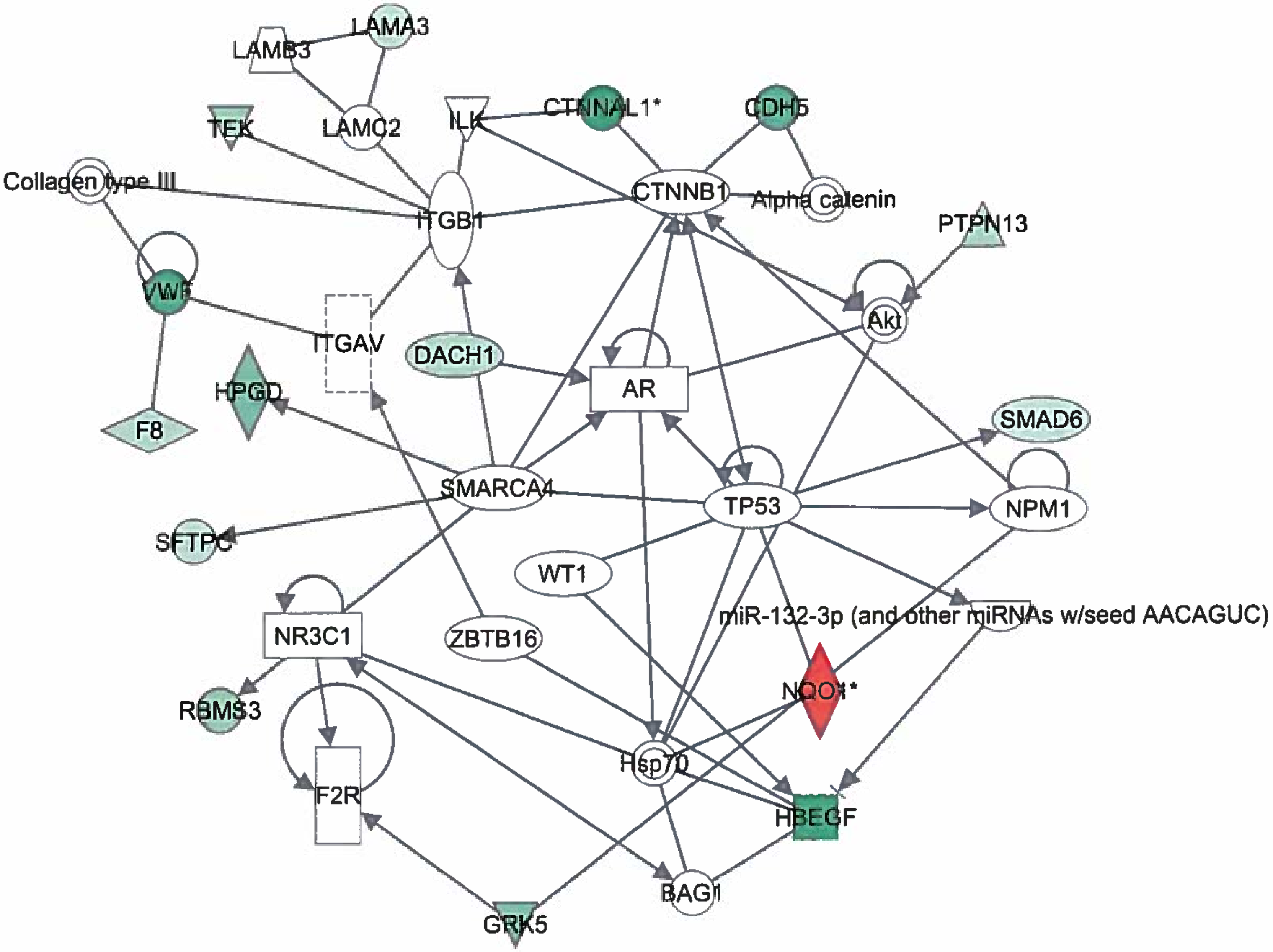

S6C

DM+DE LOCI WITHIN PROMOTERS, ADENOCARCINOMAS only
