# Supplementary material for: Genome Wide Methylome Alterations in Lung Cancer
Source: PLoS One. 2015 Dec 18;10(12):e0143826. doi: 10.1371/journal.pone.0143826 (PMC4684329; doi:10.1371/journal.pone.0143826)
Supplement: S1 File — (PDF) [file pone.0143826.s007.pdf]

Supplementary Information: Materials and Methods

All recruits were part of the pulmonary medicine and thoracic surgery practices at Albany Medical Center in Albany, NY and Albert Einstein College of Medicine/Montefiore Medical Center, Bronx, NY. The indication for lung resectional surgery was for clinical indications, under a protocol approved by Albert Einstein College of Medicine/Montefiore Medical Center, and previously by Albany Medical Center and New York State Department of Health institutional review boards. For a given specimen, DNA and RNA were extracted independently from separate tissue sections.

DNA Extraction: Approximately 3.0 ug of intact high quality genomic DNA was isolated from each sampleFrozen tissue (~50 mg) was first chopped into smaller pieces using a sterile scalpel on dry ice and then ground using a plastic pestle in the presence of cold 1x PBS. Cells were lysed by incubating with extraction buffer for 1 hour at 37oC. This was followed by overnight proteinase K digestion, phenol-chloroform extraction and 24 hours dialysis at 4oC using 0.2x SSC. DNA was recovered post dialysis, concentrated with PEG and examined for quality and quantity using agarose gel electrophoresis and spectrophotometry.

RNA isolation from macroscopic tissue samples

Approximately 50 mg to 80 mg of snap frozen lung tissue was added to a tube containing 1 ml of extraction buffer and completely homogenized. Further total RNA extraction procedures were performed using RNeasy® mini kit (Qiagen) according to the manufacturer's suggestions including an optional 30 min DNase I treatment. Total RNA was quantified using a Nanodrop Spectrophotometer 2000 (Thermo Scientific Inc., Waltham, MA, USA) and the quality confirmed on an Agilent 2100 bioanalyzer (Agilent Technologies, Santa Clara, CA, USA). A RIN (RNA integrity number) of 7 was the threshold used to proceed.

Approximately 100ng total RNA was amplified using Ambion WT Expression Kit following the manufacturer's instructions; 5.5 μg of amplification products were labeled with Affymetrix WT Terminal Labeling Kit, and then hybridized to Affymetrix HG Gene 1.0ST chips using Affymetrix hybridization and wash/stain kits, according to the manufacturer's instructions.

Technical Validation of HELP data

Three representative hypermethylated loci identified by HELP assay in the promoters of the two DARS and RGS3 in tumors were verified using the Sequenom MassARRAY EpiTYPER method (Sequenom, San Diego, CA). Approximately 500 ng genomic DNA was treated with the bisulfite reagent included in the EZ DNA Methylation Kit (Zymo Research, Orange, CA). Two amplicons were generated by PCR using tagged primers (DARS-F; GGAAGAGAGTAGTTGTAATTGAGGTTGTGATTTG; DARS-R: CAGTAATACGACTCACTATAGGGAGAAGGCTAAAACACCATAACAAAACTATTCCCT; RGS3-F2: GGAAGAGAGGGGGAATTGTAAAGATTTTTTTT; RGS3-LR2: CAGTAATACGACTCACTATAGGGAGAAGGCTCAACTACTTTTA CCCATCTCAAAAC). The reverse primers were tagged with the T7-promoter sequence for in vitro transcription. A touchdown PCR using the FastStart Taq DNA Polymerase (Roche, Mannheim, Germany) was performed, including 3 cycles of 95°C 30 s/60°C 30 s/72°C 1 min, and 37 cycles of 95°C 30 s/56°C 30 s/72° C 1 min. After treatment with alkaline phosphatase ExoSAP-IT (Affymetrix, Santa Clara, CA), the PCR products were transcribed, cleaved by RNase A at specific bases (U or C), and spotted on a 384-pad SpectroCHIP (Sequenom) followed by spectral acquisition on a MassARRAY Analyzer. The methylation calls were performed by the EpiTyper software v1.0 (Sequenom), which generates quantitative results (methyl CpG/total CpG) for each CpG site. The

methylation degree was calculated by methylated CCGG/methylated +unmethylated CCGG (Methylation ratio by rank, Y-axis). For HELP assay, the methylation degree was indicated by delta value from HpaII vs MspI (Delta value by rank, X-axis). Spearman Rank Order Correlation software was used for correlation analysis

Identification and Stability of top DM loci as T-NT classifiers:

The whole data set with mixed histology or 14 adenocarcinoma only data set was split into training and testing sets. The T and NT pairs were divided randomly into two partitions: a training set containing 2/3 of the samples (14 T and 14 NT for whole data set; 9 T and 9 NT for adenocarcinoma only data set) and a testing set containing 1/3 of the samples (7 T and 7 NT for whole data set; 5 T and 5 NT for adenocarcinoma only data set). This process was repeated 10 times. In each training set, paired t test was performed to select the most significant 100 or 25 DM loci. Then prediction models were built using sequential minimal optimization algorithm for support vector classifier[i] implemented in R package RWeka[ii,iii] on each training set and the performance evaluation was conducted on the respective testing set. The performance variables of the models were averaged across these 10 trials.

---

[i] *J. C. Platt (1998). Fast training of Support Vector Machines using Sequential Minimal Optimization. In B. Schoelkopf, C. Burges, and A. Smola (eds.), Advances in Kernel Methods – Support Vector Learning. MIT Press.*

[ii] *Kurt Hornik, Christian Buchta, Achim Zeileis (2009) Open-Source Machine Learning: R Meets Weka. Computational Statistics, 24(2), 225-232.*

[iii] *Ian H. Witten and Eibe Frank (2005). Data Mining: Practical Machine Learning Tools and Techniques. 2nd Edition, Morgan Kaufmann, San Francisco.*
