# Supplementary material for: Genome Wide Methylome Alterations in Lung Cancer
Source: PLoS One. 2015 Dec 18;10(12):e0143826. doi: 10.1371/journal.pone.0143826 (PMC4684329; doi:10.1371/journal.pone.0143826)
Supplement: S1 Table — (PDF) [file pone.0143826.s008.pdf]

Supplementary Table 1: Donor Characteristics

Serial No.	Subject ID	Tumor Histology	Age	Gender	Smoking Status	Smoking Dose (Pack Years)	Gene expression
1	538	Ad	65	0	1	30	Y
2	541	Sq	75	1	2	87	Y
3	542	Ad	65	0	1	9	Y
4	543	Ad	71	0	1	51	Y
5	551	Ad	58	1	2	60	Y
6	552	Sq	57	1	2	12	Y
7	558	Ad	81	1	1	41	Y
8	560	Ad	64	0	2	58	Y
9	561	Sq	63	1	2	35	Y
10	566	Sq	58	0	2	68	Y
11	581	Sq	46	0	1	15	Y
12	596	Sq	62	0	1	17	Y
13	603	Ad	78	0	0	0	Y
14	606	Ad	57	0	2	40	Y
15	610	Ad	75	0	1	1	Y
16	619	AdSq	77	0	1	16	Y
17	642	Ad	55	0	2	10	Y
18	653	Ad	64	0	1	20	Y
19	670	AdSq	79	0	1	51	Y
20	675	Ad	77	0	1	150	Y
21	692	Ad	62	0	2	33	Y
22	699	Ad	NA	1	0	0	N
23	713	Ad	NA	0	0	0	N
24	716	Ad	NA	0	2	54	N
