# Supplementary material for: Genome Wide Methylome Alterations in Lung Cancer
Source: PLoS One. 2015 Dec 18;10(12):e0143826. doi: 10.1371/journal.pone.0143826 (PMC4684329; doi:10.1371/journal.pone.0143826)
Supplement: S2 Table — (PDF) [file pone.0143826.s009.pdf]

Supplementary Table 2: Top 25 differentially methylated loci

(A) All histologies, promoters, (B) All histologies, gene bodies, (C) Adenocarcinomas only, promoters (D) Adenocarcinomas only, Gene Bodies

Loci previously reported as DM are denoted by a superscript to the corresponding literature reference in the gene name.

**S2A) Top 25 DM Loci across all Histologies within promoters**				
**GENE SYMBOL**	**FUNCTION**	**DELTA (log2)***	**P-VALUE**	**FDR P-VALUE**
**HYPERMETHYLATED**				
C7orf54	chromosome 7 open reading frame 54	-1.32	4.52E-07	8.00E-05
DARS	aspartyl-tRNA synthetase	-1.22	3.42E-08	4.37E-05
SPTAN1	spectrin, alpha, non-erythrocytic 1	-1.17	6.03E-06	2.37E-04
DOM3Z	dom-3 homolog Z (C. elegans)	-1.13	1.21E-04	1.36E-03
PCNX	pecanex homolog (Drosophila)	-1.13	7.59E-05	1.02E-03
CTNNAL1[19]	catenin (cadherin n-associated protein), alpha-like 1	-1.12	3.49E-05	6.32E-04
ZNF366	zinc finger protein 366	-1.1	6.56E-05	9.27E-04
CDKN2B[4, 16]	cyclin-dependent kinase inhibitor 2B (p15, inhibits CDK4)	-1.05	1.00E-04	1.21E-03
UBE2E2	ubiquitin-conjugating enzyme E2E 2	-1.03	8.70E-06	2.88E-04
VTI1A	vesicle transport through interaction with t-SNAREs homolog 1A (yeast)	-1.01	3.35E-07	7.29E-05
HIST1H1A[16, 19]	histone cluster 1, H1a	-1.01	4.98E-06	2.15E-04
TMF1	TATA element modulatory factor 1	-0.98	3.04E-06	1.68E-04
LEAP2	liver expressed antimicrobial peptide 2	-0.97	2.42E-06	1.52E-04
**HYPOMETHYLATED**				
NQO1	NAD(P)H dehydrogenase, quinone 1	1.99	6.36E-07	8.90E-05
SIRPB1[16]	signal-regulatory protein beta 1	1.88	1.22E-06	1.13E-04
UNC5CL	unc-5 homolog C (C. elegans)-like	1.8	1.73E-07	6.06E-05
NFIA	nuclear factor I/A	1.58	3.11E-04	2.53E-03
CST1	cystatin SN	1.44	1.42E-07	5.79E-05
SIRPD[16]	signal-regulatory protein delta	1.43	8.55E-07	9.86E-05
TMCO1	transmembrane and coiled-coil domains 1	1.42	4.65E-07	8.07E-05
CLDN18	claudin 18	1.39	2.73E-06	1.60E-04
KIAA1462	Junctional Protein Associated With Coronary Artery Disease	1.38	6.68E-06	2.50E-04
SLC8A3	solute carrier family 8 (sodium/calcium exchanger), member 3	1.36	7.27E-06	2.62E-04
C2orf40	chromosome 2 open reading frame 40	1.36	8.25E-07	9.73E-05
CLEC4E	C-type lectin domain family 4, member E	1.33	5.93E-07	8.69E-05

**S2B) Top 25 DM Loci across all histologies within Gene Bodies**				
**GENE SYMBOL**	**FUNCTION**	**DELTA (log2)**	**P-VALU E**	**FDR P-VALU E**
**HYPERMETHYLATED**				
NOL10	nucleolar protein 10	-2.20	6.22E-08	4.83E-05
ARHGEF12	Rho guanine nucleotide exchange factor (GEF) 12	-1.93	7.34E-08	4.98E-05

UST	uronyl-2-sulfotransferase	-1.86	2.17E-06	1.44E-04
RGS3	regulator of G-protein signaling 3	-1.85	2.43E-07	6.63E-05
MBNL2	muscleblind-like 2 (Drosophila)	-1.84	8.50E-08	5.13E-05
RXFP1	relaxin/insulin-like family peptide receptor 1	-1.81	1.53E-07	5.88E-05
CLN8	ceroid-lipofuscinosis, neuronal 8 (epilepsy, progressive with mental retardation)	-1.81	8.44E-08	5.13E-05
PASK	PAS domain containing serine/threonine kinase	-1.77	1.32E-07	5.66E-05
STARD13	StAR-related lipid transfer (START) domain containing 13	-1.77	1.21E-06	1.13E-04
PPM1H	protein phosphatase 1H (PP2C domain containing)	-1.76	8.96E-06	2.93E-04
CENPO	centromere protein O	-1.74	1.38E-07	5.72E-05
SH3BP5	SH3-domain binding protein 5 (BTK-associated)	-1.74	2.11E-07	6.36E-05
ANKS1A	ankyrin repeat and sterile alpha motif domain containing 1A	-1.74	9.91E-08	5.31E-05
**HYPOMETHYLATED**				
FBXL7	F-box and leucine-rich repeat protein 7	2.44	5.52E-08	4.81E-05
RYR2[19]	ryanodine receptor 2 (cardiac)	2.42	3.12E-08	4.36E-05
NTRK3[19]	neurotrophic tyrosine kinase, receptor, type 3	2.33	2.92E-08	4.36E-05
ADAMTS12	ADAM metallopeptidase with thrombospondin type 1 motif, 12	2.32	1.09E-06	1.09E-04
PARK2	Parkinson disease (autosomal recessive, juvenile) 2, parkin	2.3	1.92E-08	4.15E-05
FAM171A1	family with sequence similarity 171, member A1	2.28	3.02E-07	7.05E-05
PEG3	paternally expressed 3; PEG3 antisense RNA (non-protein coding); zinc finger, imprinted 2	2.24	9.06E-08	5.22E-05
TNR	tenascin R (restrictin, janusin)	2.23	2.10E-07	6.35E-05
DPP6[16, 19]	dipeptidyl-peptidase 6	2.22	9.82E-08	5.29E-05
ST6GALNAC3	ST6 (alpha-N-acetyl-neuraminyl-2,3-beta-galactosyl-1,3)-N-acetylgalactosaminide alpha-2,6-sialyltransferase 3	2.21	4.17E-08	4.53E-05
SLC8A3	solute carrier family 8 (sodium/calcium exchanger), member 3	2.15	1.30E-08	4.15E-05
BCAS3	breast carcinoma amplified sequence 3	2.14	2.76E-08	4.36E-05
**S2C) Top 25 DM Loci among Adenocarcinomas within Promoters**				
**HYPERMETHYLATED**				
RPL23AP32	ribosomal protein L23a pseudogene 32	-1.26	5.12E-05	1.10E-02
mir-26a-1	mir-26a-1	-1.23	1.00E-04	1.11E-02
SPTAN1	spectrin, alpha, non-erythrocytic 1 (alpha-fodrin)	-1.08	2.79E-04	1.25E-02
RASL12	RAS-like, family 12	-1.02	6.27E-05	1.10E-02
SLC27A6 [19, 21]	solute carrier family 27 (fatty acid transporter)	-0.96	1.57E-04	1.16E-02
KPNB1	karyopherin (importin) beta 1	-0.93	6.97E-04	1.56E-02
APIP	APAF1 interacting protein	-0.9	1.94E-03	2.26E-02
UBE2E2	ubiquitin-conjugating enzyme E2E 2	-0.89	4.74E-04	1.40E-02

GIMAP1	GTPase, IMAP family member 1	-0.88	1.41E-03	1.99E-02
GBA2	glucosidase, beta (bile acid) 2	-0.88	1.74E-05	1.10E-02
m	schlafen family member 13	-0.88	9.17E-04	1.70E-02
FIG4	FIG4 homolog (S. cerevisiae)	-0.87	1.85E-04	1.18E-02
SNRK	SNF related kinase	-0.87	2.08E-03	2.33E-02
**HYPOMETHYLATED**				
NQO1	NAD(P)H dehydrogenase, quinone 1	1.84	6.64E-05	1.10E-02
UNC5CL	unc-5 homolog C (C. elegans)-like	1.74	1.52E-05	1.10E-02
SIRPB1 [19]	signal-regulatory protein beta 1	1.73	1.86E-04	1.18E-02
DAB2	disabled homolog 2, mitogen-responsive phosphoprotein (Drosophila)	1.6	6.68E-05	1.10E-02
GIMAP5	GTPase, IMAP family member 5	1.5	4.14E-06	1.10E-02
SCN4A	sodium channel, voltage-gated, type IV, alpha subunit	1.48	1.47E-05	1.10E-02
DAB2	disabled homolog 2, mitogen-responsive phosphoprotein (Drosophila)	1.45	5.88E-04	1.49E-02
OVCH1	ovochymase 1	1.31	2.65E-04	1.24E-02
MRPL38	mitochondrial ribosomal protein L38	1.29	4.58E-05	1.10E-02
MAEL	maelstrom homolog (Drosophila)	1.27	2.89E-03	2.68E-02
IGKC	immunoglobulin kappa constant	1.21	3.57E-05	1.10E-02
RHOH	ras homolog family member H	1.13	6.61E-04	1.54E-02
**S2D) Top 25 DM Loci among Adenocarcinomas within Gene Bodies**				
**HYPERMETHYLATED**				
AKAP13	A kinase (PRKA) anchor protein 13	-1.57	1.56E-04	1.16E-02
ANKRD44	ankyrin repeat domain 44	-1.61	6.10E-04	1.50E-02
ANKS1A	ankyrin repeat and sterile alpha motif domain containing 1A	-1.52	2.02E-04	1.19E-02
CCDC92	coiled-coil domain containing 92	-1.56	1.87E-05	1.10E-02
GALNT1	(GalNAc-T1) galactosamine:polypeptide N-acetylgalactosaminyltransferase 1 (GalNAc-T1)	-1.64	7.76E-05	1.10E-02
LRRFIP1	leucine rich repeat (in FLII) interacting protein 1	-1.5	2.03E-04	1.19E-02
MEIS2	Meis homeobox 2	-1.62	2.26E-05	1.10E-02
PRKCE	protein kinase C, epsilon	-1.52	3.21E-04	1.28E-02
RNF144B	ring finger protein 144B	-1.53	3.02E-04	1.27E-02
ROS1	c-ros oncogene 1 , receptor tyrosine kinase	-1.58	1.60E-04	1.16E-02
RXFP1	relaxin/insulin-like family peptide receptor 1	-1.65	1.24E-04	1.12E-02
SLC7A1	solute carrier family 7 (cationic amino acid transporter, y+ system), member 1	-1.52	1.25E-04	1.13E-02
SPTBN1	spectrin, beta, non-erythrocytic 1	-1.55	2.45E-05	1.10E-02
**HYPOMETHYLATED**				
FAM171A1	family with sequence similarity 171, member A1	2.22	3.49E-05	1.10E-02
FBXL7	F-box and leucine-rich repeat protein 7	2.13	1.12E-04	1.12E-02
PARK2	Parkinson disease (autosomal recessive, juvenile) 2, parkin	2.12	2.19E-05	1.10E-02
BCAS3	breast carcinoma amplified sequence 3	2.09	3.39E-05	1.10E-02
RYR2	ryanodine receptor 2 (cardiac)	2.07	1.63E-04	1.16E-02
CNTNAP5 [16, 21]	contactin associated protein-like 5	1.98	3.04E-05	1.10E-02
RHOJ [19]	ras homolog gene family, member J	1.97	4.93E-05	1.10E-02
PRKCQ	protein kinase C, theta	1.95	4.26E-04	1.36E-02

ZNF423	zinc finger protein 423	1.93	1.71E-04	1.17E-02
TNR	tenascin R (restrictin, janusin)	1.93	2.72E-04	1.24E-02
CDH13 [4, 19, 21]	cadherin 13, H-cadherin (heart)	1.93	1.09E-05	1.10E-02
TPK1	thiamin pyrophosphokinase 1	1.92	5.44E-05	1.10E-02

*DELTA: (log base 2) is the magnitude of differential methylation in T vs NT; negative implies hypermethylation
