# Supplementary material for: Genome Wide Methylome Alterations in Lung Cancer
Source: PLoS One. 2015 Dec 18;10(12):e0143826. doi: 10.1371/journal.pone.0143826 (PMC4684329; doi:10.1371/journal.pone.0143826)
Supplement: S3 Table — (PDF) [file pone.0143826.s010.pdf]

Supplementary Table 3: Methylation-Expression overlay.

Genes also identified as top 25 in the Methylation-alone analysis are denoted by an asterisk (*). Genes previously reported as DM as well as DE are denoted by a superscript to the corresponding reference in the gene name.

Supplementary Table 3A TOP 25 DIFFERENTIALLY METHYLATED LOCI IN PROMOTERS IN THE VICINITY OF DE GENES

GENE SYMBOL	FUNCTION	DELTA* Log2	P-VALUE	FDR P-VALUE	GENE EXP
**HYPERMETHYLATED**					
HBEGF	heparin-binding EGF-like growth factor	-0.91	1.78E-05	4.27E-04	DOWN
DPT	dermatopontin	-0.84	1.19E-05	3.41E-04	DOWN
AGER	advanced glycosylation end product-specific receptor	-0.8	1.90E-04	1.83E-03	DOWN
SPARCL1[*,19]	SPARC-like 1 (hevin)	-0.8	3.24E-05	6.05E-04	DOWN
PTPRM	protein tyrosine phosphatase, receptor type, M	-0.75	8.78E-05	1.11E-03	DOWN
ARHGEF6	Rac/Cdc42 guanine nucleotide exchange factor (GEF) 6	-0.75	7.08E-06	2.58E-04	DOWN
TMEM88	transmembrane protein 88	-0.71	3.99E-05	6.84E-04	DOWN
SEMA6A	sema domain, transmembrane domain (TM), and cytoplasmic domain, (semaphorin) 6A	-0.67	1.75E-04	1.73E-03	DOWN
HPGD	hydroxyprostaglandin dehydrogenase 15-(NAD)	-0.63	8.92E-05	1.12E-03	DOWN
PEBP4	phosphatidylethanolamine-binding protein 4	-0.6	1.32E-04	1.44E-03	DOWN
RBMS3	RNA binding motif, single stranded interacting protein	-0.57	6.47E-04	4.17E-03	DOWN
FILIP1	filamin A interacting protein 1	-0.56	4.59E-05	7.45E-04	DOWN
CCBE1	collagen and calcium binding EGF domains 1	-0.53	3.97E-04	2.98E-03	DOWN
**HYPOMETHYLATED**					
NQO1[*]	NAD(P)H dehydrogenase, quinone 1	1.99	6.36E-07	8.90E-05	UP
CST1[*]	cystatin SN	1.44	1.42E-07	5.79E-05	UP
TNS4	tensin 4	0.92	1.12E-05	3.31E-04	UP
FUT2	fucosyltransferase 2 (secretor status included)	0.9	8.79E-05	1.11E-03	UP
MELK[*]	maternal embryonic leucine zipper kinase	0.81	2.11E-03	9.65E-03	UP
FAM83A[*,19]	family with sequence similarity 83, member A	0.81	9.49E-05	1.17E-03	UP
MMP9[*]	matrix metallopeptidase 9 (gelatinase B, 92kDa gelatinase, 92kDa type IV collagenase)	0.8	1.29E-03	6.79E-03	UP
SLCO1B3	solute carrier organic anion transporter family, member 1B3	0.79	8.81E-04	5.17E-03	UP
AKR1B10	aldo-keto reductase family 1,	0.79	6.95E-04	4.38E-03	UP

	member B10 (aldose reductase); aldo-keto reductase family 1, member B10-like				
ABCA12	ATP-binding cassette, sub-family A (ABC1), member 12	0.69	5.61E-04	3.78E-03	UP
KRT15	keratin 15	0.56	1.24E-04	1.38E-03	UP
TYMS	thymidylate synthetase	0.47	5.85E-04	3.89E-03	UP
**S3B) TOP 25 DIFFERENTIALLY METHYLATED LOCI IN GENE BODIES IN THE VICINITY OF DE GENES**					
**HYPERMETHYLATED**					
FERMT1	fermitin family homolog 1 (Drosophila)	-0.89	1.01E-05	3.12E-04	UP
SLC7A5	solute carrier family 7 (cationic amino acid transporter, y+ system), member 5	-0.88	6.98E-06	2.56E-04	UP
FAP	fibroblast activation protein, alpha	-0.86	6.06E-04	3.99E-03	UP
KRT15	keratin 15	-0.82	1.99E-04	1.88E-03	UP
ETV4	ets variant 4	-0.79	1.84E-06	1.34E-04	UP
TFAP2A*	transcription factor AP-2 alpha (activating enhancer binding protein 2 alpha)	-0.78	3.62E-05	6.45E-04	UP
TPX2	TPX2, microtubule-associated, homolog (Xenopus laevis)	-0.74	2.08E-04	1.94E-03	UP
FOXM1	forkhead box M1	-0.74	1.43E-05	3.77E-04	UP
AFAP1	actin filament associated protein 1	-0.71	7.30E-06	2.62E-04	UP
SIX1	SIX homeobox 1	-0.7	6.67E-04	4.26E-03	UP
GREM1*	gremlin 1, cysteine knot superfamily, homolog (Xenopus laevis)	-0.69	4.18E-04	3.09E-03	UP
AURKA	aurora kinase A; aurora kinase A pseudogene 1	-0.68	7.44E-04	4.59E-03	UP
TYMS	thymidylate synthetase	-0.66	5.92E-08	4.83E-05	UP
**HYPOMETHYLATED**					
AGBL1	ATP/GTP binding protein-like 1	2.11	9.75E-07	1.03E-04	DOWN
RHOJ[19]	ras homolog gene family, member J	2.06	1.69E-07	6.04E-05	DOWN
LDB2	LIM domain binding 2	1.94	4.45E-08	4.60E-05	DOWN
GHR	growth hormone receptor	1.94	4.94E-08	4.72E-05	DOWN
ITGA8*[19]	integrin, alpha 8	1.92	1.30E-07	5.65E-05	DOWN
ABCB1	ATP-binding cassette, sub-family B (MDR/TAP), member 1	1.86	2.93E-08	4.36E-05	DOWN
SEMA5A	sema domain, seven thrombospondin repeats (type 1 and type 1-like), transmembrane domain (TM) and short cytoplasmic domain, (semaphorin) 5A	1.86	1.28E-05	3.55E-04	DOWN
GPM6A	glycoprotein M6A	1.85	5.18E-07	8.31E-05	DOWN
GRIA1*[19]	glutamate receptor, ionotropic, AMPA 1	1.8	3.22E-06	1.73E-04	DOWN
PTPRM	protein tyrosine phosphatase,	1.76	1.02E-06	1.05E-04	DOWN

	receptor type, M				
COL29A1	collagen, type XXIX, alpha 1	1.76	1.90E-07	6.19E-05	DOWN
SLIT3	slit homolog 3 (Drosophila)	1.68	4.22E-07	7.85E-05	DOWN
**S3C) TOP 25 DIFFERENTIALLY METHYLATED LOCI IN PROMOTERS IN THE VICINITY OF DE GENES (WITHIN ADENOCARCINOMAS ONLY)**					
**HYPERMETHYLATED**					
RPL23AP32*	ribosomal protein L23a pseudogene 32	-1.26	5.12E-05	1.10E-02	DOWN
CTNNAL1*[19]	catenin (cadherin-associated protein), alpha-like 1	-0.82	7.08E-03	4.17E-02	DOWN
C6orf155		-0.81	4.65E-03	3.36E-02	DOWN
HBEGF	heparin-binding EGF-like growth factor	-0.80	2.26E-03	2.41E-02	DOWN
TMEM88	transmembrane protein 88	-0.78	1.82E-04	1.17E-02	DOWN
CASP12	caspase 12	-0.73	9.58E-05	1.11E-02	DOWN
FILIP1	filamin A interacting protein 1	-0.73	5.15E-06	1.10E-02	DOWN
VWF	Coagulation Factor VIII	-0.69	3.93E-03	3.10E-02	DOWN
**CDH5**	cadherin 5, type 2	-0.65	3.47E-05	1.10E-02	DOWN
ITGA8*,[19]	integrin, alpha 8	-0.65	8.36E-03	4.56E-02	DOWN
ARHGAP29	Rho GTPase activating protein 29	-0.64	9.35E-03	4.84E-02	DOWN
CLEC3B	C-type lectin domain family 3, member B	-0.62	7.00E-04	1.57E-02	DOWN
CRTAC1	cartilage acidic protein 1	-0.61	7.53E-03	4.31E-02	DOWN
GPR126	G-Protein Coupled Receptor 126	-0.60	1.43E-03	2.00E-02	DOWN
PTPRM	protein tyrosine phosphatase, receptor type, M	-0.60	3.52E-03	2.94E-02	DOWN
**HYPOMETHYLATED**					
NQO1	NAD(P)H dehydrogenase, quinone 1	1.84	6.64E-05	1.10E-02	UP
LOC651751		1.21	3.57E-05	1.10E-02	UP
IGKC // IGKC	immunoglobulin kappa constant	1.21	3.57E-05	1.10E-02	UP
CST1	cystatin SN	1.11	1.90E-04	1.18E-02	UP
XAGE1D	Cancer/Testis Antigen 12.1	1.02	4.59E-03	3.34E-02	UP
AIM2	Interferon-Inducible Protein and putative tumor suppressor	0.74	3.72E-03	3.02E-02	UP
MMP11	matrix metallopeptidase 11	0.64	3.38E-03	2.88E-02	UP
LGR4	Leucine-Rich Repeat Containing G Protein-Coupled Receptor 4	0.55	8.17E-03	4.50E-02	UP
PVRL4	Nectin involved in cell adhesion	0.42	6.03E-03	3.84E-02	UP
EFNA4	EPH-Related Receptor Tyrosine Kinase Ligand	0.20	2.61E-03	2.56E-02	UP
**S3D) TOP 25 DIFFERENTIALLY METHYLATED LOCI IN GENE BODIES IN THE VICINITY OF DE GENES (WITHIN ADENOCARCINOMAS ONLY)**					
CP	Ceruloplasmin (Ferroxidase)	-1.08	2.32E-04	1.77E-02	UP
FAP	fibroblast activation protein, alpha	-1.04	8.23E-04	1.37E-03	UP
C6orf125		-1.00	4.99E-04	6.65E-06	UP
NLN	Neurolysin (Metallopeptidase)	-0.98	7.67E-05	1.32E-05	UP
TPX2	TPX2, microtubule-associated, homolog (Xenopus laevis)	-0.93	1.58E-05	2.00E-05	UP
KIF26B	kinesin family member 26B	-0.90	4.34E-04	1.12E-05	UP
SLC7A5	solute carrier family 7 (amino acid transporter light chain, L system),	-0.85	8.50E-03	2.31E-05	UP

	member 5				
GPR39	G protein-coupled receptor 39	-0.79	1.76E-04	6.93E-04	UP
NELL1	Neural Epidermal Growth Factor-Like	-0.74	3.73E-03	4.60E-02	UP
GREM1[*]	gremlin 1, cysteine knot superfamily	-0.74	5.07E-04	6.25E-04	UP
CDH3	cadherin 3, type 1	-0.74	5.48E-03	1.30E-02	UP
GFRA3	Glial Cell Line-Derived Neurotrophic Factor Receptor	-0.74	8.13E-05	3.01E-02	UP
SIX1	SIX homeobox 1	-0.74	2.22E-03	6.59E-03	UP
**HYPOMETHYLATED**					
RHOJ[19]	ras homolog gene family, member J	1.97	4.93E-05	3.93E-07	DOWN
CDH13 [4, 19, 21]	cadherin 13, H-cadherin (heart)	1.93	1.09E-05	2.24E-04	DOWN
TSPAN18	transmembrane 4 superfamily	1.77	2.00E-04	8.54E-06	DOWN
PDE1C	phosphodiesterase 1C, calmodulin-dependent	1.74	8.12E-05	1.44E-04	DOWN
ANO2	anoctamin 2 Transmembrane Protein 16B	1.74	7.31E-05	6.22E-07	DOWN
INMT	Indolethylamine N-Methyltransferase	1.69	6.56E-05	1.46E-06	DOWN
PLCB4	phospholipase C, beta 4	1.68	1.78E-03	6.65E-05	DOWN
LDB2	LIM domain binding 2	1.65	1.51E-04	3.93E-07	DOWN
GRIA1[*, 19]	glutamate receptor, ionotropic, AMPA 1	1.64	3.33E-04	1.87E-06	DOWN
SLIT3	slit homolog 3 (Drosophila)	1.63	7.44E-05	1.68E-04	DOWN
ITGA8[*, 19]	integrin, alpha 8	1.61	1.59E-04	3.92E-05	DOWN
GHR	growth hormone receptor	1.59	5.45E-05	9.02E-06	DOWN

DELTA(log2) is the magnitude of differential methylation (HpaII vs MspI in T vs NT)
