# Supplementary material for: Genome Wide Methylome Alterations in Lung Cancer
Source: PLoS One. 2015 Dec 18;10(12):e0143826. doi: 10.1371/journal.pone.0143826 (PMC4684329; doi:10.1371/journal.pone.0143826)
Supplement: S4 Table — (PDF) [file pone.0143826.s011.pdf]

Supplementary Table 4. Primers used.

Gene	Primer	Size (bp)
HBEGF	forward: TTGTGCTCAAGGAATCGGCT	114
	reverse: CAACTGGGGACGAAGGAGTC	
TMEM88	forward: GCCTGCGCTGTTCTTGTAAC	100
	reverse: CAACTGGGGACGAAGGAGTC	
CASP12	forward: GGACCAAGCACTGGGATCAA	201
	reverse: GCAAGAGCCGACCATGAGTA	
FILIP1	forward: CCTGGTGCAAGCAAAGTGAC	83
	reverse: CCTGACACTGACTGGGTTCC	
VWF	forward: GTGTGTCCGAGTGAAGGAGG	117
	reverse: CAGCACGCTGAGGTCTTACA	
NQO1	forward: GGATTGGACCGAGCTGGAAA	199
	reverse: CAAACTGAAACACCCAGCCG	
CST1	forward: GTCCGCTGCGGGTACTAA	191
	reverse: GTTCTCCCAGGGAACTTCGT	
XAGE1D	forward: CCGGCGTCAAGGTGAA	111
	reverse: ACCAGCTTGCGTTGTTTCAG	

Quantitative real-time RT-PCR (RT-qPCR) for the most dysregulated genes was performed with Power SYBR Green PCR master mix in a 96-well optical plate using an ABI 7500 real-time PCR system (all from Life Technologies, Carlsbad, CA). All RT-qPCR primers and product size are listed above. The reaction condition was 95C 10min, then 95C, 15s, 64C, 20s, 72C, 45s for 45 cycles.The qPCR was performed in technical triplicates for each sample. Additional ddH2O blank and RNA without reverse transcriptase samples served as negative controls for each gene. Melting analysis for one additional cycle was performed. Where necessary, an RNA-specific strategy that avoids contaminating genomic DNA amplification and false positives was used
