# Supplementary material for: Genome Wide Methylome Alterations in Lung Cancer
Source: PLoS One. 2015 Dec 18;10(12):e0143826. doi: 10.1371/journal.pone.0143826 (PMC4684329; doi:10.1371/journal.pone.0143826)
Supplement: S5 Table — (PDF) [file pone.0143826.s012.pdf]

Supplementary Table 5. Promoter CGI and CGS distinction in DMxDE analysis

Data Set	Compartment	# DM loci within 2 kb of DE genes	# loci associated with Hypermethylation and Downregulation of corresponding gene	# loci associated with Hypomethylation and Upregulation of corresponding gene	% Loci
All Histologies	CG Islands	158	6	0	4
	CG Shores	289	6	1	2
Adenocarcinomas	CG Islands	107	11	0	11
	CG Shores	213	5	0	3

Methylation x Expression relationships were examined in DM loci within CG islands and CG shores. A small percentage of DM loci within CGI and CGS demonstrated canonical relationships between DM and DE.
