# Supplementary material for: Genome Wide Methylome Alterations in Lung Cancer
Source: PLoS One. 2015 Dec 18;10(12):e0143826. doi: 10.1371/journal.pone.0143826 (PMC4684329; doi:10.1371/journal.pone.0143826)
Supplement: S6 Table — (PDF) [file pone.0143826.s013.pdf]

Supplementary Table 6: Summary of IPA Analyses

Histology	Compartment	Change	# loci	# Genes	# Genes associated with Cancer	B-H adj p-value of enriched canonical pathway	DM Loci forming hubs/nodes in cancer-related network
ALL	PR	DM	1300	1079	313	>0.05	NA
ALL	GB	DM	42109	4968	1480	0.000000149*	DNMT1, DNMT3A, CDKN2B, CDH1 (Hypermethylated)
ADENO	PR	DM	1670	1150	324	>0.05	NA
ADENO	GB	DM	40136	4163	1179	0.0152*	LRRK2, SVIL, AR, (Hypomethylated) CAST, MCM2 (Hypermethylated)
ALL	PR	DM+DE	51	37	20	>0.05	NA
ALL	GB	DM+DE	2244	220	104	>0.05	NA
ADENO	PR	DM+DE	118	71	34	0.000157**	NQO1 (Hypomethylated) VWF, HBEGF, DACH1, (Hypermethylated)
ADENO	GB	DM+DE	3598	302	128	>0.05	NA

* Molecular Mechanisms of Cancer

** Axonal Guidance Signaling
