# Supplementary material for: Genome Wide Methylome Alterations in Lung Cancer
Source: PLoS One. 2015 Dec 18;10(12):e0143826. doi: 10.1371/journal.pone.0143826 (PMC4684329; doi:10.1371/journal.pone.0143826)
Supplement: S7 Table — (PDF) [file pone.0143826.s014.pdf]

Supplementary Table 7. Discrimination Stability of DM loci set

Table 7A: Whole data set top 100 DM loci classification results for each testing set.

Iteration #	True Positive	True Negative	False Negative	False Positive	Sensitivity	Specificity	Accuracy
1	6	6	1	1	0.8571	0.8571	0.8571
2	5	6	2	1	0.7143	0.8571	0.7857
3	6	6	1	1	0.8571	0.8571	0.8571
4	6	6	1	1	0.8571	0.8571	0.8571
5	7	7	0	0	1.0000	1.0000	1.0000
6	7	7	0	0	1.0000	1.0000	1.0000
7	5	7	2	0	0.7143	1.0000	0.8571
8	5	7	2	0	0.7143	1.0000	0.8571
9	3	7	4	0	0.4286	1.0000	0.7143
10	6	7	1	0	0.8571	1.0000	0.9286
Average					0.8000	0.9429	0.8714

Table 7B: Whole data set top 25 DM loci classification results for each testing set

Iteration #	True Positive	True Negative	False Negative	False Positive	Sensitivity	Specificity	Accuracy
1	5	7	2	0	0.7143	1.0000	0.8571
2	4	7	3	0	0.5714	1.0000	0.7857
3	7	7	0	0	1.0000	1.0000	1.0000
4	6	6	1	1	0.8571	0.8571	0.8571
5	7	7	0	0	1.0000	1.0000	1.0000
6	6	7	1	0	0.8571	1.0000	0.9286
7	5	7	2	0	0.7143	1.0000	0.8571
8	6	7	1	0	0.8571	1.0000	0.9286
9	6	7	1	0	0.8571	1.0000	0.9286
10	5	7	2	0	0.7143	1.0000	0.8571
Average					0.8143	0.9857	0.9000
